# ncOrtho: efficient and reliable identification of miRNA orthologs

**DOI:** 10.1093/nar/gkad467

**Published:** 2023-06-01

**Authors:** Felix Langschied, Matthias S Leisegang, Ralf P Brandes, Ingo Ebersberger

**Affiliations:** Applied Bioinformatics Group, Institute of Cell Biology and Neuroscience, Goethe University, Frankfurt, Germany; Institute for Cardiovascular Physiology, Goethe University, Frankfurt, Germany; German Center of Cardiovascular Research (DZHK), Partner site RheinMain, Frankfurt, Germany; Institute for Cardiovascular Physiology, Goethe University, Frankfurt, Germany; German Center of Cardiovascular Research (DZHK), Partner site RheinMain, Frankfurt, Germany; Applied Bioinformatics Group, Institute of Cell Biology and Neuroscience, Goethe University, Frankfurt, Germany; Senckenberg Biodiversity and Climate Research Centre (S-BIK-F), Frankfurt am Main, Germany; LOEWE Centre for Translational Biodiversity Genomics (TBG), Frankfurt am Main, Germany

## Abstract

MicroRNAs (miRNAs) are post-transcriptional regulators that finetune gene expression via translational repression or degradation of their target mRNAs. Despite their functional relevance, frameworks for the scalable and accurate detection of miRNA orthologs are missing. Consequently, there is still no comprehensive picture of how miRNAs and their associated regulatory networks have evolved. Here we present ncOrtho, a synteny informed pipeline for the targeted search of miRNA orthologs in unannotated genome sequences. ncOrtho matches miRNA annotations from multi-tissue transcriptomes in precision, while scaling to the analysis of hundreds of custom-selected species. The presence-absence pattern of orthologs to 266 human miRNA families across 402 vertebrate species reveals four bursts of miRNA acquisition, of which the most recent event occurred in the last common ancestor of higher primates. miRNA families are rarely modified or lost, but notable exceptions for both events exist. miRNA co-ortholog numbers faithfully indicate lineage-specific whole genome duplications, and miRNAs are powerful markers for phylogenomic analyses. Their exceptionally low genetic diversity makes them suitable to resolve clades where the phylogenetic signal is blurred by incomplete lineage sorting of ancestral alleles. In summary, ncOrtho allows to routinely consider miRNAs in evolutionary analyses that were thus far reserved to protein-coding genes.

## Graphical Abstract

**Graphical Abstract:**
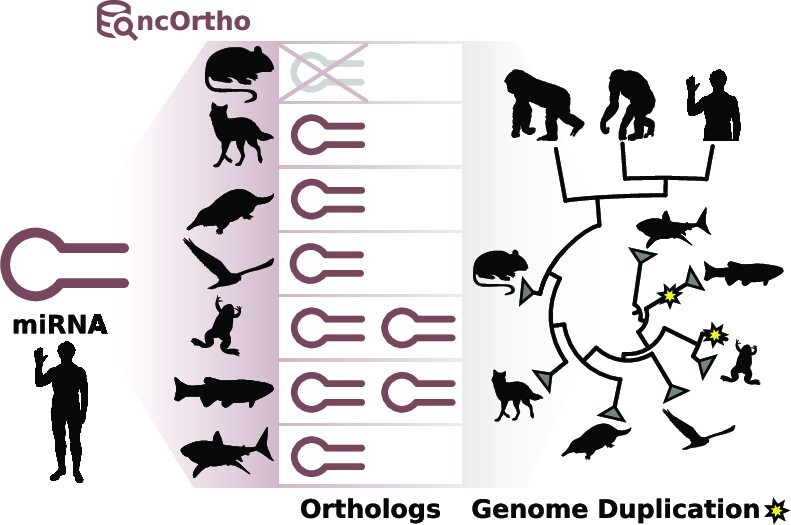

## INTRODUCTION

MicroRNAs (miRNAs) are single-stranded, short non-coding RNAs of ∼22 nucleotides (nt) in length that have essential roles in fine-tuning the expression network of eukaryotic cells (1–3). Canonical miRNAs are transcribed by RNA Polymerase II resulting in primary miRNAs (pri-miRNA) that form at least one distinctive hairpin structure. Pri-miRNA are cleaved by the Microprocessor complex, which contains Drosha and DiGeorge syndrome critical region 8, resulting in microRNA precursors (pre-miRNA) (4). Pre-miRNAs are approx. 55–70 nt long sequences, which are exported into the cytoplasm by Exportin 5 where the terminal loop of the pre-miRNAs is removed to create miRNA duplexes (4,5). This duplex is subsequently resolved, and one of the two strands is loaded into an Argonaut protein to form the mature RNA-induced silencing complex (RISC) (6,7). Target mRNAs are silenced post-transcriptionally through binding of the RISC, which is induced by a pairing of the 7 nt long seed region in the mature miRNA and a 6–8 nt long target site in the mRNA (8).

miRNA-dependent gene regulation in animals dates at least back to the last common ancestor of the Eumetazoa that lived more than 800 million years ago (9,10). The acquisition of novel miRNA families in the course of evolution has been correlated with body-plan innovations and an increase of overall morphological complexity (11–14). Novel miRNAs can emerge via the repurposing of already transcribed sequences, such as parts of introns or protein-coding exons (15,16). Once these miRNAs are integrated into a regulatory network, purifying selection contributes to their preservation (11). Still, findings that suggest the loss of evolutionary old and hence well integrated miRNA families were presented (17,18), but these were contrasted by the claim that the loss of established miRNAs is exceedingly rare (19,20). It was argued that false-positive miRNA annotations in the miRBase repository (21), which served as the basis of the analyses, paired with a limited sensitivity of the miRNA homolog search created a spurious signal of miRNA loss (22). Meanwhile the manually curated MirGeneDB has put miRNA-based research on a more solid foundation (19). In this database, each miRNA entry is supported by corresponding transcript data and meets stringent annotation criteria. Moreover, MirGeneDB embeds miRNA annotations into an evolutionary context, and version 2.1 of the database provides information about the representation of miRNA genes across 75 species (23). While this increases the specificity of miRNA annotations, MirGeneDB faces two main challenges: first, miRNAs that are expressed at low levels or under specific conditions are prone to be missed. Second, the taxonomic resolution and therefore the evolutionary information content in the data will remain low because the requirement of multi-tissue transcriptomic datasets to support miRNA calls renders the integration of novel taxa into MirGeneDB cost- and labor-intensive. For many protected or rare species, it is even virtually impossible to gather the necessary data, particularly if their export falls under international regulations for species transfer in the context of CITES (https://cites.org/eng/prog/Permit_system).

Scanning genome sequences for the presence of orthologs to miRNAs in MirGeneDB offers a powerful alternative. It allows to propagate miRNA annotation across species independent from the availability of transcriptome data. Genome sequences abound in public databases and can be obtained even with non-invasive sampling (e.g. 24,25). Thus, large and evolutionary diverse taxon collections can be analyzed, which is crucial for improving the signal-to-noise ratio in evolutionary analyses (26). For protein-coding genes, the identification of orthologs and the generation of comprehensive phylogenetic profiles, i.e. the presence/absence pattern of orthologs across large taxon sets, is well established (e.g. 27). In the case of miRNAs, the tools available remain sparse. Individual solutions for genome-wide scans for miRNA homologs have been proposed, but published approaches either lack publicly available software implementations (e.g. 17,28), or are limited to pre-defined taxon sets (e.g. MapMi; 29). To our knowledge, the only currently available method to identify miRNA orthologs in custom assemblies requires the alignment of whole genome sequences (30). While this approach is straightforward, in principle, the computational overhead is immense. For example, an alignment of 242 placental mammalian genomes took two months of computation time on the Amazon Cloud utilizing 260 instances, each equipped with 32 virtual CPUs (31). Moreover, aligning whole genomes of species covering the full diversity of vertebrates is difficult (32). Therefore, only a small fraction of the genome sequences that are currently being generated (e.g. 32–34) will be considered in whole genome alignments.

To close this methodological gap, we have developed ncOrtho, the first software that facilitates a targeted search for miRNA orthologs in individual genome sequences. ncOrtho scales linearly in time with both the number of miRNAs and the number of taxa included in the ortholog search, and therefore enables miRNA ortholog searches in large and customizable genome collections. ncOrtho identifies orthologs with high sensitivity and specificity irrespective of the genome annotation status. The resulting phylogenetic profiles provide the basis for studying miRNA evolution at an unprecedented scale and represent the first step towards projecting regulatory networks of miRNAs from model- to non-model organisms.

## MATERIALS AND METHODS

### Data

Genome assemblies of 161 mammalian species, 241 non-mammalian vertebrates, and 16 invertebrate species were downloaded from NCBI Refseq Genomes release 207 (33; Supplementary Table S1). Locations and pre-miRNA sequences for all 556 human miRNAs were downloaded from MirGeneDB v2.0. The MirGeneDB nomenclature was derived from miRBase (19,34), and mapping between the nomenclatures is available on the MirGeneDB webpage. Whole genome alignments were downloaded from the 100 vertebrate alignment track in the UCSC Genome Browser (35).

### Covariance models

Pairwise orthology assignments between protein-coding genes were identified with OMA standalone v2.4.1 (36) and served as anchors to identify shared syntenic regions between the genomes of humans and each core species. Positional miRNA orthologs were identified in the shared syntenic regions as specified in the main text and were used to generate and train a Covariance Model (CM) for each human miRNA. Sequences in the individual training sets were aligned with R-Coffee (37) and were annotated with secondary structure information calculated by RNAalifold from the Vienna RNA package 2.0 (38). The multiple sequence alignment together with the secondary structure information were then used as input for the Infernal package to train and calibrate the corresponding CM in default settings (39). CM-based searches were performed with the *cmsearch* algorithm provided in the Infernal v1.1 package, and search results were filtered for sequences that achieve at least 50% of the maximally achievable bit score (i.e. the score of the reference miRNA given the CM).

### Evaluation of orthology assignment performance

MirGeneDB provided the gold-standard to evaluate orthology assignments by ncOrtho (9). For individual species, MirGeneDB used genome assemblies other than those deposited in NCBI RefSeq Genome. In these cases, we mapped the miRNAs stored in MirGeneDB to the NCBI RefSeq assembly of the corresponding species using BLASTn (cutoff: ≥90% identity and ≥80% query coverage) (40). During the software benchmark, we compared the location of each predicted miRNA ortholog to the genomic location of MirGeneDB entries from the same miRNA family. In case of an overlap, the candidate was accepted as a true positive (TP). Results that cover a genomic region with no matching entry in MirGeneDB were tentatively assigned as false positives (FP). An assignment was considered as false negative (FN) if the corresponding MirGeneDB entry was not identified as an ortholog. All cases in which no ortholog was detected and MirGeneDB has no entry for that family and species were counted as true negatives (TN). We then calculated the sensitivity as TP/(TP + FN), the specificity as TN/(TN + FP), the accuracy as (TP + TN)/(TP + FP + FN + TN), and the *F*1-score as (2 × TP)/(2 * TP + FP + FN).

### Alternative approaches of miRNA ortholog identification

Whole Genome alignments: We downloaded the alignment blocks of the 100 Vertebrate alignment from the UCSC Browser FTP-server (http://hgdownload.soe.ucsc.edu/goldenPath/hg38/multiz100way/maf), and extracted the longest alignment block covering each human pre-miRNA locus annotated in MirGeneDB (9). We considered an ortholog candidate to be found in a species if its sequence covered at least 70% of the human miRNA locus excluding gaps. BLASTn search: We used the pre-miRNA sequences from MirGeneDB as input for a local BLASTn search (40) using the ‘blastn’ task with default parameter settings. The best hit was used as the miRNA ortholog candidate. MapMi: Mature miRNA sequences provided by MirGeneDB were used as input for the MapMi implementation on the EMBL-EBI webserver using the 59 ‘Ensembl Species’ set (41,42).

### Population variation analysis

Human single nucleotide polymorphism (SNP) data was retrieved from dbSNP build 153 (43). We considered only data from gnomAD v2.1 Genomes, which contains variants from 15708 human genomes (44), to ensure comparability between the SNP counts for the following seven genomic regions: (i) mature, (ii) star and (iii) loop region of a miRNA, the 30 nt (iv) upstream- and (v) downstream flanking regions that are annotated by MirGeneDB, (vi) all CDS and (vii) all lncRNA genes of the GRCh38 assembly. SNP counts for each region were normalized by their respective length and then multiplied by 1000 resulting in the SNP density per 1kb.

### Phylogenetic analyses

The presence/absence patterns of human miRNAs were visualized using PhyloProfile (45) once on the level of individual genes and once on the miRNA family level. The evolutionary emergence of a miRNA family was tentatively dated to the last common ancestor of the two most distantly related species in which an ortholog from the corresponding family was detected. miRNA orthologs were aligned with MUSCLE v3.8.155 (46). The individual alignments were concatenated and alignment columns with more than 50% gaps were removed. ModelFinder (47) identified the TIM3 + F + R5 evolutionary model as the best fitting model for the data, and the maximum likelihood tree was calculated using IQ-TREE v1.6.8 with 1000 ultra-fast bootstrap replicates (48,49). The full pipeline used for reconstructing pre-miRNA sequence trees is implemented into the ncOrtho package. Phylogenetic trees were visualized and annotated with iTOL (50) or the ETE 3 toolkit (51). Competing phylogenetic hypothesis testing was performed with the Approximately Unbiased (AU) test as implemented in IQ-TREE (49).

## RESULTS

miRNAs are not yet routinely considered in large-scale evolutionary analyses because scalable approaches for the reliable identification of miRNA orthologs across large and phylogenetically diverse taxon collections were missing. We therefore developed ncOrtho to identify orthologs of miRNA genes in genome assemblies irrespective of their annotation status. Key to this approach is the use of Covariance Models (CMs), which are probabilistic models that integrate the consensus nucleotide sequence with the secondary structure of a functional RNA and facilitate a sensitive and discriminative homolog identification (52,53). The phylogenetic profiles generated by ncOrtho provide detailed insights into the taxonomic distribution, the minimal evolutionary age, and the duplication/deletion history of the analyzed miRNAs.

### Algorithm

**Figure 1.**
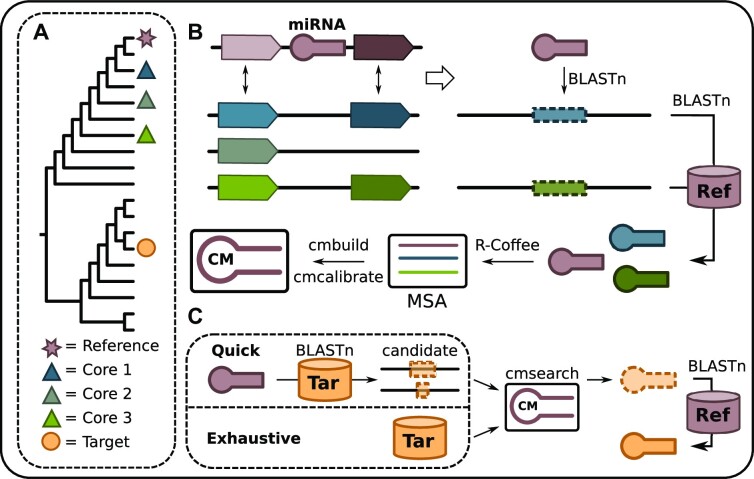
Workflow of ncOrtho. (**A**) Taxon selection. The species that harbors the miRNA in focus is selected as ‘reference’. ‘Core species’ are used to identify positional miRNA orthologs for Covariance Model (CM) training. The CM-based miRNA ortholog search is performed in the ‘target’ species. (**B**) Covariance Model training. Positional miRNA ortholog candidates are identified via BLASTn in regions of conserved protein-coding gene order. Candidates are confirmed as orthologs if a reverse BLASTn search in the reference genome (Ref) identifies the reference miRNA as best hit. Positional miRNA orthologs are aligned, and the resulting multiple sequence alignment (MSA) is then used for CM training. (**C**) Targeted ortholog search. In ‘quick mode’, the reference miRNA is used as a query for a BLASTn search against the target genome (Tar) to narrow the search space to candidate regions of ∼2000 bp in length. In the exhaustive mode, the entire genome is used. A cmsearch using the pre-trained CM identifies then putative miRNA orthologs. Candidate orthologs are confirmed if a BLASTn search in the reference genome obtains the reference miRNA as the best hit.

ncOrtho accepts an individual pre-miRNA (the ‘reference miRNA’) together with its genomic position in the reference species as input. Additionally, two lists of taxa together with the corresponding genome sequences are required. The first list defines a set of ‘core species’ that are considered in the training phase of the covariance model (Figure 1A). Core species should be closely related enough for microsynteny to be conserved to an extent that a comprehensive identification of positional miRNA orthologs is possible. Additionally, the last common ancestor of the core species should not be older than the miRNA gene of interest. To facilitate core species selection, we supply a function with which microsynteny conservation can be estimated as part of the ncOrtho package (Supplementary Figure S1). For the core species, the annotation of all protein-coding genes as well as the pairwise orthology assignments to the proteins encoded in the reference species are needed. The second list specifies the target species whose genomes should be scanned for the presence of miRNA orthologs. ncOrtho then follows a two-stage procedure to accomplish the ortholog search (see Figure 1). Initially, a set of high confidence orthologs is compiled from the core species which are then used for CM construction and training. Subsequently, the CM is utilized in the search for miRNA orthologs in all target species.

#### Covariance model training

ncOrtho uses a set of high confidence orthologs to the reference miRNA for CM construction. This training data is compiled by exploiting collinearity of homologous genomic segments (shared synteny) to identify positional miRNA orthologs in the core species. We distinguish two cases: If the miRNA is positioned between two protein-coding genes, we use the flanking genes as syntenic anchors and consider a region in the core species’ genome as shared syntenic if no more than *k* protein-coding genes separate the orthologs of the anchor genes (parameter –mgi; Default: *k* = 3). To account for gene losses or gene annotation artefacts, we provide the option to consider up to *n* flanking genes as alternative syntenic anchors (parameter –max_anchor_dist; Default: *n* = 1) (Figure 1B). If the miRNA is located within a protein-coding gene, the shared syntenic region in the core species is the genomic locus harboring the ortholog of the protein-coding gene. If no ortholog could be detected, the algorithm proceeds as if the miRNA-gene would be in an intergenic region. Upon the identification of a shared syntenic region, its sequence is extracted and a reciprocal best BLASTn hit approach using the reference pre-miRNA sequence as query identifies a miRNA ortholog, if present (54). This procedure is repeated for all core species, which results in a set of positional orthologs that are used to train a covariance model of the reference miRNA.

#### Targeted miRNA ortholog search using covariance models

The genome sequence of each target species is scanned for the presence of a miRNA using the corresponding CM (Figure 1C). Candidates represented by CM search hits meeting the score threshold (see methods) are accepted as an ortholog if a BLASTn search against the reference genome using the candidate as query reveals the reference miRNA as the best hit. If two or more candidate orthologs for the same reference miRNA are confirmed by the reverse search, all are kept as co-orthologs. The runtime complexity of CM searches renders scans of entire genomes across larger taxon sets time consuming (Supplementary Figure S2). Therefore, ncOrtho provides the option to reduce the search space by first searching for subsequences in the target genome that share a local sequence similarity to the reference miRNA (‘Quick’ mode; Figure 1C). Hits are then extended by 1000 nucleotides (nt) up- and downstream, and the resulting candidate regions serve as input for a subsequent refined search using the reference miRNA-specific CM.

### Benchmarking ncOrtho orthology assignments

#### Performance

To evaluate ncOrtho, we used the manually curated miRNA database MirGeneDB as a gold standard (19). We identified orthologs to 556 miRNA genes representing 266 miRNA families. *Homo sapiens* served as the reference species, and *Macaca mulatta, Gorilla gorilla*, *Pongo abelii* and *Nomascus leucogenys* were used as core species. 242 miRNA genes were located within, and 314 between protein-coding genes, and there was no difference in the core ortholog set sizes between the two groups (Supplementary Figure S3). For the benchmark, we concentrated on 20 vertebrate species that were represented both in MirGeneDB 2.0 and in the RefSeq partition of the NCBI genome database. We further included 16 invertebrate animals to sound out the sensitivity limits of ncOrtho in miRNA families that are conserved across the Bilateria (9). Table 1 shows that sensitivity and specificity of ncOrtho in Quick mode are very high across all analyzed vertebrates. Using this mode, ncOrtho identifies orthologs with a median runtime of 0.7 s per miRNA and species (Supplementary Figure S4). In the invertebrates, orthologs are identified with near perfect specificity but the sensitivity drops substantially. Repeating the ortholog search in ‘Exhaustive’ mode (see Figure 1C) had almost no effect on the sensitivity, but the run time increased by two orders of magnitude (Table 1, Supplementary Figure S2). Thus, the search heuristic used in the Quick mode of ncOrtho does not account for the sensitivity drop. On closer inspection, we noticed that the missing of invertebrate miRNA orthologs coincides with changes in the loop and star region that are specific to the invertebrate orthologs (Supplementary Figure S5).

**Table 1.** Performance of ncOrtho. Median runtime per species

Taxon set	Setting	Accuracy	*F*1-score	Specificity	Sensitivity	Runtime (median)
Vertebrates	Quick	0.93	0.93	0.90	0.97	27 min
	Exhaustive	0.93	0.93	0.90	0.97	35 h 57 min
Invertebrates	Quick	0.93	0.24	0.99	0.14	7 min
	Exhaustive	0.93	0.26	0.99	0.15	6 h 13 min

#### ncOrtho orthologs without MirGeneDB confirmation

**Figure 2.**
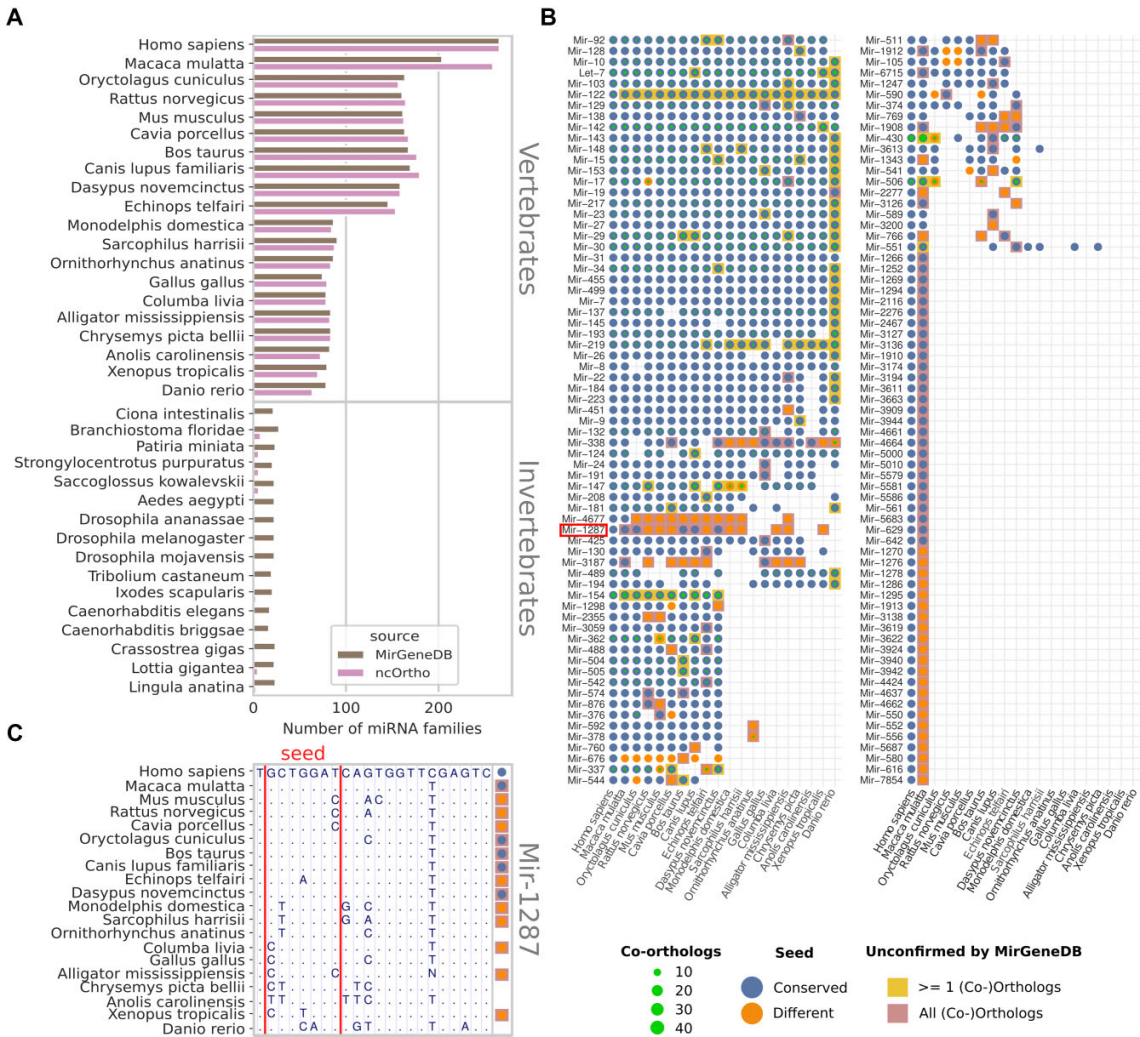
Orthology-based phylogenetic profiles of human miRNAs. (**A**) Human miRNA families represented in 35 species according to MirGeneDB 2.0 and ncOrtho, respectively. (**B**) Phylogenetic profiles for 138 miRNA families with one or more orthologs unconfirmed by MirGeneDB across 20 vertebrate species. The presence of a miRNA ortholog in a species is indicated by a dot. Dot color: blue – seed sequence is identical to the human miRNA; orange – seed sequence differs. The green inlay indicates the presence of co-orthologs, and the inlay diameter is proportional to the co-ortholog numbers summed over all family members. Cell color indicates the fraction of (co-)orthologs that are supported by MirGeneDB: white – all; yellow – some; red – none. A 20-species alignment of the genomic region harboring the mature Mir-1287 (highlighted in red) is shown in (**C**). Only nucleotides that differ from the human reference are specified. The seed region of the miRNA is marked with red lines, and a dot next to a sequence indicates that an ortholog to Mir-1287 was detected in this species where the color coding follows (B). Note, the ortholog assignment uses the full pre-miRNA sequence and not only the section shown in the alignment.

ncOrtho identifies miRNA orthologs with an overall high accuracy, and the mature human miRNAs align with <3 mismatches to 99% of all identified orthologs (Supplementary Figure S6A). Additionally, there was no difference in the predicted secondary structure between miRNA orthologs identified by ncOrtho and those deposited in MirGeneDB (Supplementary Figure S6B). This indicates that ncOrtho fulfills the criteria for miRNA ortholog annotation defined by the miRNA community (34). Still, on first sight a specificity of 0.90 in the vertebrate set of target species suggests that the false positive rate can be improved. Interestingly, the fraction of orthologs predicted solely by ncOrtho is the highest in rhesus macaque (Figure 2A). Humans and rhesus have a genome-wide average pairwise sequence identity of 93% (55), which should render the identification of miRNA orthologs straightforward. We therefore used this species as an example to show that our findings can be at least partly explained by missing data in MirGeneDB.

A parsimonious interpretation of the phylogenetic profiles of human miRNA orthologs as provided by MirGeneDB v2.0 suggested two independent losses of miRNA families in rhesus and 55 gains on the human lineage (Figure 2B). Complementing the profiles with orthologs exclusively predicted by ncOrtho reduced the number of human-specific families that are confined to humans to 0, and many of the ncOrtho-only miRNA orthologs share the seed sequence with the human miRNA (Figure 2B). Moreover, the updated version 2.1 of MirGeneDB now includes 16 miRNA families from 8 species that were predicted by us but were not represented in MirGeneDB v2.0, for example Mir-6715 or Mir-1912 (Supplementary Figure S7). Thus, many ncOrtho-exclusive orthologs, which were initially considered as false positive assignments in our benchmark, represent genuine orthologs that are not represented in MirGeneDB v2.0. The specificity of ncOrtho is therefore substantially higher than 0.91. However, there are also cases where ncOrtho extends the phylogenetic profile with entries that are not covered by MirGeneDB. For example, Mir-1287 is annotated as human-specific even in MirGeneDB v2.1, but ncOrtho identified orthologs with conserved seed sequences in species as distant as the armadillo (*D. novemcinctus;* Figure 2C*)*. Consulting the 100 vertebrate whole genome alignment (35) revealed that the orthologs identified by ncOrtho reside in a genomic region that is conserved across the vertebrates.

#### Comparison of ncOrtho with other tools

To the best of our knowledge, only three tools have been developed that allow the identification of miRNA homologs in genome assemblies. Of these, we did not consider miRNAminer (56) because it does not support batch requests for multiple miRNAs and/or species. A second tool, miROrtho (28), is no longer publicly available. MapMi (29) detects putative miRNA homologs in a fixed set of 59 species, of which 18 are also represented in MirGeneDB 2.0. We further compared the performance of ncOrtho to the identification of miRNA orthologs using the 100 Vertebrate whole genome alignment (see Supplementary Figure S8 for further details) and using a naïve BLASTn search. Applying the same benchmark criteria as described above (see Table 1), revealed that ncOrtho outperforms the alternative methods by far (Table 2). Only BLASTn was superior in terms of sensitivity, but this comes at the cost of a specificity of only 0.37.

**Table 2.** Performance of ncOrtho and of three alternative approaches in the identification of human miRNA orthologs across 18 vertebrate species. The highest score is highlighted in bold format

Tool	Accuracy	*F*1-score	Specificity	Sensitivity
ncOrtho	**0.93**	**0.93**	**0.91**	0.95
MapMi	0.55	0.50	0.64	0.46
WGA	0.80	0.78	0.86	0.71
BLASTn	0.66	0.73	0.37	**0.97**

#### Runtime analysis

The run time of the ncOrtho search scales linear with both the number of reference miRNAs and the number of target species (Supplementary Figure S9). The ortholog search of 556 human miRNAs took 5 h and 23 min with 4 CPUs in rhesus (*Macaca mulatta*). ncOrtho evaluated 8490 genomic regions resulting in 1222 ortholog candidates that entered the final validation. The same search using mouse as target species finished after 53 min (Supplementary Figure S2). Here, numbers drop to only 2481 genomic regions and 638 ortholog candidates, which explains the shorter run time. Note that much of the surplus of candidates in rhesus results from few miRNAs that are in repeat rich regions. For example, the ortholog search for Mir-1271 alone requires the evaluation of 2498 genomic regions in rhesus. The median search time for orthologs to 556 human miRNAs across all 402 vertebrate species was 23 min when using 4 CPUs (59 min CPU time).

### Phylogenetic profile of human miRNA orthologs across 402 vertebrates

**Figure 3.**
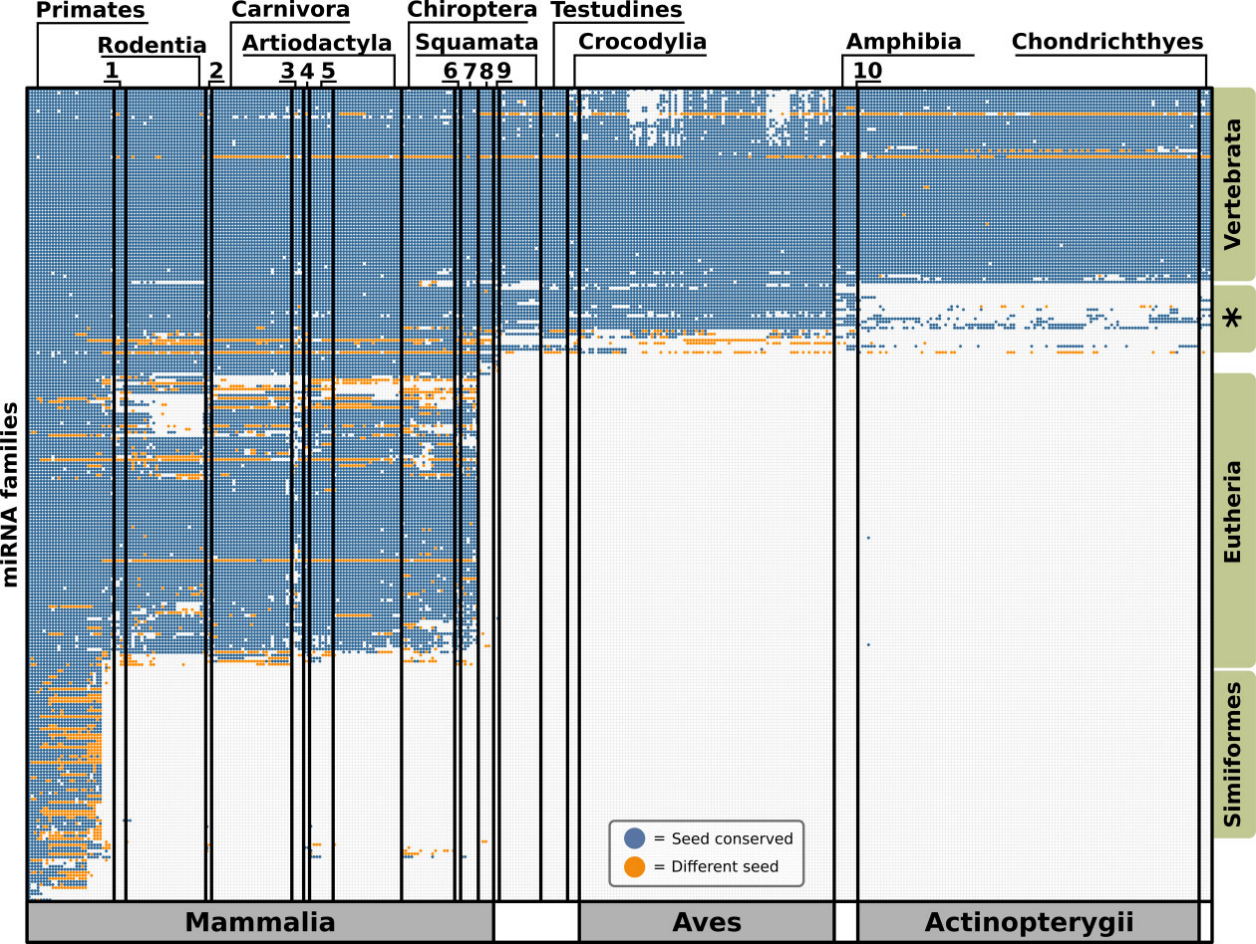
Phylogenetic profiles of 266 human miRNA families across 402 vertebrates. The representation of a miRNA family (rows) by at least one ortholog in a species (column) is indicated by a dot. Species are ordered according to increasing taxonomic distance to humans. Green labels indicate the taxonomic distribution of the corresponding miRNA families. The asterisk indicates human miRNAs whose orthologs are either confined to the *Sarcopterygii* or occur only sporadically in more distantly related taxa. 1 = Lagomorpha, 2 = Scandentia/Dermoptera, 3 = Eulipotyphla, 4 = Pholidota, 5 = Perissodactyla, 6 = Xenarthra, 7 = Afrotheria, 8 = Metatheria, 9 = Monotremata, 10 = *Latimeria chalumnae*.

Investigating the evolutionary trajectory of miRNAs has so far been hindered by a limited taxon sampling in publicly available data repositories of miRNA orthologs. In many cases, systematic groups up to the order level are represented by only one or at most a few representatives. When analyzing missing miRNAs, this makes it hard to differentiate between noise, e.g. due to incomplete data, and a true miRNA loss. To obtain a better resolved picture of miRNA evolution, we extended the taxon sampling for the miRNA ortholog search to represent 402 vertebrate genomes. The resulting phylogenetic profiles summarized on the miRNA family level are shown in Figure 3 and the gene-level resolution is shown in Supplementary Figure S10. The genomic locations and sequences of all ncOrtho orthology assignments are provided in Supplementary Table S2.

#### the evolutionary age of human miRNA families

The profiles reveal that the 266 human miRNA families can be broadly distinguished into four phylostratigraphic layers (53; see Supplementary Table S3). 53 families are confined to the higher primates (Simiiformes) and 86 families are represented only in the Eutheria. The emergence of 71 families predates the diversification of vertebrates. Eighteen miRNA families are preferentially found in the sarcopterygians (tetrapods and coelacanth). However, some of these families are also sporadically present in representatives of the earlier branching lineages, which makes their evolutionary age harder to assess. We next overlaid the phylogenetic profiles of the miRNA families with information about the conservation of the human seed sequence (see Figure 3). In the evolutionarily older miRNAs, the seed sequences are overall highly conserved. This picture changes substantially for the miRNA families that emerged in the last common ancestor of the higher primates. Here, seed sequence changes are commonly observed, and in many cases a single substitution suffices to explain the differences between the individual primate lineages (Supplementary Figure S11).

#### Loss of miRNA families

Our results show that the loss of miRNA families is rare. The entire data matrix in Figure 3 has only 12.1% missing data, i.e. a miRNA family was not detected in a species that diversified after the evolutionary emergence of the family (Supplementary Table S3; see Supplementary Figure S10 for a gene level resolution). In most cases, these are sporadic absences of miRNA families in individual species. Without further manual curation, this is best interpreted as noise. However, a few notable exceptions exist where the absence of a miRNA family is consistently observed in several species within a systematic group, which results in the characteristic ‘windows’ in the phylogenetic profiles (see Figure 3). We can differentiate two main scenarios. The joint loss of several miRNA families in the rodents is one prominent example for a concerted loss of several miRNA families in the last common ancestor of a monophyletic group of species. The situation is different for the missing miRNA families in birds. Integrating the presence/absence pattern of miRNA families with the evolutionary relationships of the birds indicates multiple and independent losses of the same miRNA family on individual evolutionary lineages (Supplementary Figure S12).

#### Whole genome duplications extend the miRNA repertoire

**Figure 4.**
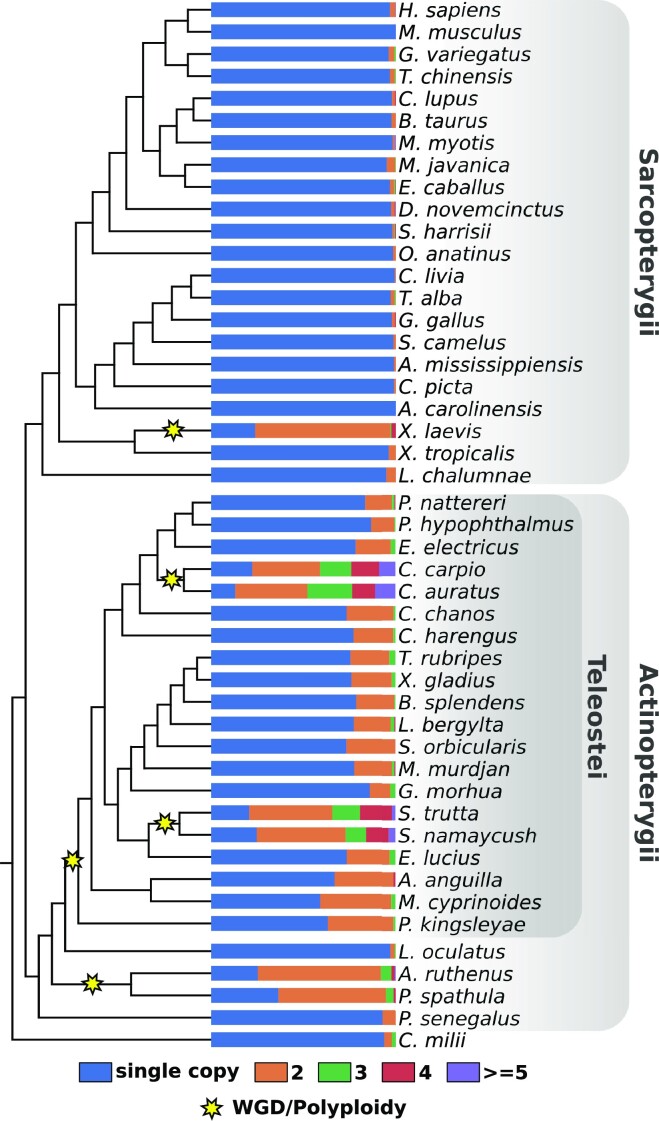
miRNA co-ortholog counts flag whole genome duplications. For each species, the fraction of human miRNAs that are represented by a single ortholog, or by two or more co-orthologs is shown. Yellow star indicates whole genome duplication events (WGD) or polyploidization (see main text). Cypriniformes are represented by *C. carpio* and *C. auratus*, and Salmoniformes are represented by *S. trutta*, and *S. namaycush*. Results for the full taxon set are shown in Supplementary Figure S13.

Lineage-specific duplications of a gene results in two copies that are both co-orthologous to the corresponding gene in a species that branched of prior to the duplication event (57). The number of co-orthologs is therefore correlated with the number of successive lineage-specific duplications. ncOrtho detected miRNAs represented by two or more co-orthologs in almost all taxa (Figure 4; see Supplementary Figure S10 for the full taxon set). However, the fraction of miRNAs with co-orthologs varies, in parts, substantially between species. Within the tetrapods and coelacanth (Sarcopterygii), co-orthologs are overall rare with one exception: in the African clawed frog (*Xenopus laevis*) about three quarters of the represented human miRNA genes have two co-orthologs. The ray-finned fish (Actinopterygii) are substantially more diverse. Co-orthologs are rare in the early branching lineages, except for the Acipenseriformes represented by the sterlet (*Acipenser ruthenus*) and the paddle fish (*Polyodon spathula*). Throughout the teleosts, the fraction of human miRNAs represented by two or more co-orthologs are up to 15 times higher compared to the tetrapods. On most lineages, human miRNA genes are represented by two co-orthologs, but three or four co-orthologs are common (>10% for either class) in both the Salmoniformes and the Cypriniformes. Reconciling the lineage-specific increase of co-ortholog numbers with existing evidence for polyploidization or whole genome duplications (WGD) results in a perfect match (see Figure 4). Two primordial diploid frog species likely hybridized giving rise to the allotetraploid *X. laevis* (58). Polyploidizations were reported for the Acipenseriformes (59,60), a teleost-specific whole genome duplication was proposed (3R-Hypothesis; 61-62), and one additional round of WGD (4R) occurred in the Salmoniformes and in the Cypriniformes, respectively (63–65).

### Phylogenomics with miRNA genes

The targeted search for miRNA orthologs across vertebrate diversity has reconstructed the evolutionary trajectory of human miRNAs at an unprecedented scale and resolution. Individual studies based on limited data have explored the use of miRNAs for the reverse approach, where patterns of sequence change between miRNA orthologs should inform about the evolutionary histories of the species they reside in (e.g. 22,61,66). We next investigated the potential of miRNAs for resolving evolutionary relationships in greater detail. Phylogenetic markers should meet two criteria: they should be rarely lost to warrant taxon-gene matrices with little missing data. Moreover, their within-species diversity should be low over evolutionary time scales to reduce the risk that incomplete lineage sorting (ILS) of ancestral alleles blurs the phylogenetic signal generated by speciation events (67). Since miRNAs fulfill the first requirement (see Figure 3) we next investigated their diversity.

#### Genetic diversity of miRNA genes

**Figure 5.**
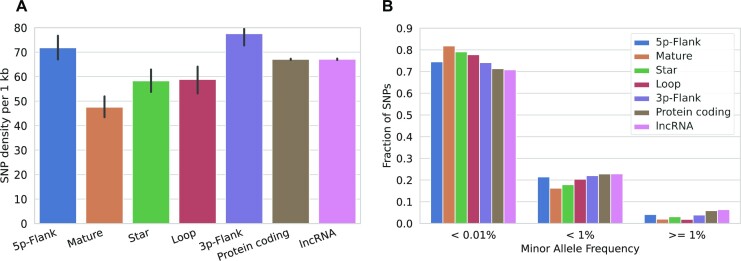
Population-level variation of different regions in the human genome. (**A**) Mean and variance of the SNP density for various regions in the human genome. ‘mature’, ‘star’ and ‘loop’ denote the corresponding parts of a miRNA gene. 5p- and 3p-Flank represents the 30 nt flanking region of miRNA genes on either side. (**B**) Mean fraction of SNPs from (A) with rare (<0.01%), uncommon (<1%), and common (≥1%) minor allele frequencies.

We determined the frequency of SNPs in human miRNA genes separately for the mature, star and loop regions and compared this to the SNP frequency in flanking regions of the miRNA, in protein-coding sequences, and in long non-coding RNAs. This revealed a significantly lower frequency in the miRNA regions compared to the other regions in the human genome (*t*-test, *P*-value < 10^−8^; Figure 5A), with the mature miRNA having the lowest diversity among all. Moreover, the represented variants have a lower minor allele frequency (Figure 5B). Both observations are in line with constraints on the evolutionary change of miRNAs that are imposed by miRNA secondary structure formation and by their function. The sequence of the mature miRNA must remain conserved since it mediates the pairing to the target mRNA (8). This constraint extends to the star sequence as it must form a stem–loop during pre-miRNA hairpin formation although individual mismatches are admissible (34). A reduced diversity compared to other genes is therefore a feature that most likely applies to miRNAs in general, irrespective of the species they reside in.

#### Vertebrate tree of life reconstructed with pre-miRNA sequences

**Figure 6.**
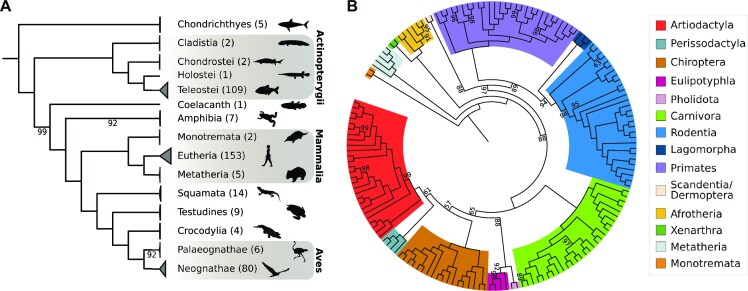
Maximum likelihood phylogeny of the vertebrates using pre-miRNA sequences. The two trees were reconstructed from a miRNA supermatrix comprising 400 species and 12 618 alignment columns. Branch labels denote percent bootstrap support, and only values <100 are provided. (**A**) The backbone phylogeny of the vertebrates rooted with the Chondrichthyes as outgroup. The number of species represented in the collapsed clades are given in parentheses. The fully expanded tree is given as Supplementary Figure 15. (**B**) shows the mammalian subtree.

We have shown that miRNAs are rarely lost, and that their genetic diversity is lowest among all investigated genomic regions. The orthology-assignments of ncOrtho for 556 human miRNAs across 402 vertebrate species comprises therefore an unprecedented data basis for miRNA-based phylogenomics. Figure 6 shows the maximum likelihood tree that is based on a supermatrix compiled from this data. *Petromyzon marinus* was not considered in this analysis because <20% of human miRNAs were represented by an ortholog in this species. Additionally, the tarsier (*Carlito syrichta)* was excluded because this species could not be stably placed in the tree (see Supplementary Figure S14). The resulting tree topology reproduces the accepted branching patterns of all major vertebrate groups (Figure 6A). This underlines that the phylogenetic signal in concatenated miRNA genes is sufficient to resolve deep splits in the vertebrate phylogeny accurately and unambiguously.

The mammalian subtree is shown in Figures 6B. Deep splits in this tree are well resolved, and monophyletic Xenarthra and Afrotheria are placed as the earlies branching lineage within the Eutheria (ML_BS_ = 100). While this resembles the Atlantogenata hypothesis (see also 68), one of the two competing hypotheses (Xenarthra branched off first) cannot be rejected with this data (p-AU = 0.06; 69). Within the Eutheria, the tree is well resolved on the order level, and here in particular for clades in which ILS is known or at least suspected to interfere with the species tree reconstruction (68,70–73) (Figure 6B, Supplementary Figure S16). The hamster-like (Hystricomorpha) and mouse-like (Myomorpha) rodents are placed in a monophyletic clade to the exclusion of the squirrel-like (Sciuromorpha) rodents (ML_BS_ = 94%; Supplementary Figure S16B). Within the Caniformia, we find support for the Ursoidea and Musteloidea families as their respective closest relatives to the exclusion of the Pinnipedia (Bootstrap support – 97%, Supplementary Figure S16C). However, for both cases, alternative topologies (see 73,74) could not be rejected (p-AU of 0.69 and 0.57, respectively). The situation is different for more recent splits. Maximal bootstrap support was obtained for monophyletic chimpanzees and bonobos as the closest living relatives of humans. Within the new world monkeys, the data supports monophyletic *Callithrix* and *Aotus* instead of placing *Aotus* in the clade formed by *Saimiri*, *Sapajus*, and *Cebus* as it was suggested by 75. For both clades, the competing hypotheses could be rejected (p-AU of 0.004 and 0.005, respectively; Supplementary Figures S16 and E).

## DISCUSSION

The accurate detection of orthologs across large taxon collections is one cornerstone of comparative genomics. It forms the foundation for tracing genes, their functions, and the corresponding functional networks across species and through time (76). ncOrtho allows to extend such analyses routinely to miRNA genes by using covariance models (CMs) in the targeted search for orthologs. Several methods attempted to extend such analyses to miRNA genes (28,29,56). Common to all approaches is the use of an uni-directional sequence similarity-based search for identifying miRNA homolog candidates, followed by a candidate curation using secondary structure- and sequence conservation filters. These tools are either no longer available or suffer from a high false-positive and false-negative rate (see Table 2). ncOrtho is the first tool to identify miRNA orthologs using a reciprocal sequence similarity search. The secondary structure constraints and primary sequence consensus of a miRNA family are captured in the CMs that are used in the ortholog search (52). The performance of these models essentially depends on the quality of the training data, and ncOrtho addresses this issue by exploiting the conservation of gene order to identify positional orthologs (77) that are then used for model training. While this warrants a highly reliable training data, it also implies that, in principle, whole genome alignments may be directly used for the identification of miRNA orthologs (30). However, this hinges on the condition that the genomic position of a miRNA genes does not change. Additionally, the phylogenetic distances between species under study must not be too large, because otherwise sequences become too diverged for aligning anything but protein coding exons (78). Eventually, the generation of whole genome alignments is computationally highly demanding, which hinders the inclusion of novel species and therefore leaves the data basis of such approaches considerably rigid. More specifically, for large collections of assemblies that are phylogenetically as diverse as the vertebrates, whole-genome alignments are not feasible (32). ncOrtho, in turn is a highly flexible framework for the accurate identification of miRNA orthologs across extensive custom taxon sets, which include species as diverse as humans and sharks who last shared a common ancestor about 600 million years ago (79).

Orthology assignment is an evolutionary reconstruction problem, and as such a ground truth does not exist. Therefore, the benchmarking of orthology assignments for protein-coding genes relies on a standardized framework that allows to assess and compare the performance of individual ortholog search tools (80). Since there is no such framework for miRNA genes yet, we have used the miRNA families that are represented in the manually curated MirGeneDB as a bona fide gold standard. Due to its stringent annotation criteria, this database was excellent for assessing the sensitivity of ncOrtho. The attempt to benchmark the specificity of the orthology assignments indicated, however, that MirGeneDB is likely not fully comprehensive and lacks an unknown but probably non-negligible number of miRNA orthologs. (23). Obviously, this does not rule out that ncOrtho may also identify miRNA orthologs that are no longer functional or makes individual spurious orthology assignments. Such cases may be characterized by multiple independent variations as for example seen in Mir-1287 (Figure 2C). Here, a thorough integration of miRNA orthology assignments based on ncOrtho paired with a subsequent curation via targeted search for these orthologs in RNAseq data would allow to trace miRNAs across species with the highest confidence. This combined approach allows to direct the miRNA search in transcriptomes to predicted, yet missing miRNAs. This would increase the probability to also detect those miRNAs that are only lowly expressed or that are expressed only under certain conditions.

ncOrtho performs a targeted ortholog search resulting in orthology assignments for pairs of species. This has several advantages. The ortholog search can use any reference sequence to start the ortholog search. This makes it independent from pre-compiled catalogs of miRNA genes and allows the tracing of both novel mirRNA genes but also the analysis of miRNAs specific to taxa that are not represented in the public miRNA databases. In the same context, ncOrtho uses a reciprocal hit criterion for the ortholog identification, instead of relying on pre-computed bit-score thresholds (81). This is particularly important, when the training data for computing the bit score thresholds does not cover the full diversity of the miRNAs. The linear scaling in time and CPU usage facilitates an ortholog search across taxon set sizes that are too resource-demanding for more complex search algorithms (82). Lastly, ncOrtho can identify orthologs in target species independent of any a-priori gene annotation. This aspect is particularly important because the annotation of miRNA genes thus far depends on the availability of deep transcriptomic sequencing of small transcripts (21). Consequently, there are substantially varying levels of miRNA annotation quality between species as a result of a dataset-availability bias (18).

While the annotation status of the target genomes does not impact the performance of ncOrtho, the assembly quality does. The number of false negative orthology assignments of genome-based searches is bound to increase when using low-coverage genome assemblies (83). In line with this hypothesis, several evolutionarily old miRNA families show a patchy presence-absence pattern of bird orthologs which could only be explained by the same miRNA being lost multiple times independently (see Figure 3 and Supplementary Figure S12). Among all analyzed vertebrates, this is a unique observation, which hints towards issues with the assembly qualities for these species. However, with the increasing number of chromosomally complete reference assemblies (84–86), the issue of genome completeness can be expected to play only a subordinate role in the future.

The phylogenetic profiles of 556 human miRNAs representing 266 miRNA families across 402 vertebrate species represent the highest resolving analysis of miRNA evolution to date. The resulting presence/absence patterns are consistent with previous studies that describe a ‘burst-wise’ acquisition of novel miRNA families in the Eutheria and Amniota (13,87,88). Here, we could pinpoint another surge of miRNA innovation to the higher primates (Simiiformes), which has been previously ascribed to either the primates or the old-world monkeys (17,20). This differentiation is important for reconstructing the genetic basis of primate diversification. But it is also essential for applications that, for example, determine the organismal origin of small RNA-seq samples (89). Next to lineage-specific gains of miRNA families, ncOrtho allows to trace likely changes in the regulatory network of miRNAs due to either miRNA loss or seed change. In the context of protein-coding genes, concerted lineage-specific loss is often interpreted as an indicator for a functional integration of the affected genes (26,90–91). Seed-pairing is the most important determinant for the targeting specificity of miRNAs (8). Notably, we find evidence for pronounced lineage-specific changes in the seed sequence, preferentially for evolutionarily young miRNAs that emerged in the last common ancestor of the higher primates. It is tempting to speculate that these seed changes contribute to the re-wiring of still flexible regulatory networks by changing the spectrum of target genes. We can now identify such events with a resolution that is unprecedented for miRNAs, which lays the foundation for investigating their relevance for re-wiring the regulatory network of miRNAs in the future.

Next to the functional implications in the context of gene regulation, changes in miRNAs allow to trace also evolutionary events on a genome-wide level. miRNAs have recently been used to investigate whole genome duplication (WGD) events in transcriptomic data from comparatively small taxon sets (92,93). Here, we have shown that co-orthologs detected by ncOrtho faithfully trace whole genome duplications across diverse collections of genome assemblies. This makes it possible to rapidly scan the plethora of vertebrate genomes that will emerge from the ongoing sequencing initiatives for indications of whole genome duplications (84,85).

miRNA sequences have been previously proposed as good phylogenetic markers (19,20), and miRNAs have been used in individual and small-scale phylogenomic studies to shed light on the evolution of individual vertebrate lineages (e.g. 68,94). Here, we could show that miRNAs fulfill two main requirements for phylogenetic markers: They are very rarely lost, and they display among all investigated regions in the human genome the lowest genetic diversity. The latter finding complements previous results that attested miRNAs a low diversity in humans, mice and pigs, but did not put these numbers in relation to other regions in the human genome (95–98). With the help of ncOrtho, the compilation of miRNA-based phylogenomic datasets is now straightforward, which allows to establish miRNAs as an alternative marker to protein-coding genes. We could show that the phylogenetic signal in miRNAs suffices to resolve even deep splits in the vertebrate tree unambiguously and with high confidence. For two recently diverged clades whose resolution was hindered by incomplete lineage sorting, we found unequivocal support for one topology. This lends support to the view that miRNAs are indeed suitable for overcoming the effect of ILS. Despite these encouraging results, trees computed from miRNA alignments also need to be treated with a grain of salt. In line with a previous report (68), our miRNA-based phylogeny of the mammals agrees with the Atlantogenatha hypothesis. However, a topology test reveals that the alternative Xenarthra hypothesis does not explain the data significantly worse. This can indicate either a limited phylogenetic signal in the data, or alternatively that hybridization blurred the phylogenetic signal at the onset of eutherian diversification (99–101). In the light of our results, incomplete lineage sorting becomes a less likely explanation.

In summary, miRNAs are essential regulators of gene expression and their tracing across a comprehensive taxon collection is bound to shed light on the emergence and evolution of the underlying regulatory networks. At the same time, miRNAs are highly informative with respect to the evolution of the species they reside in. With the help of ncOrtho the potential of miRNAs for addressing both questions can now be tapped on a comprehensive scale independent of pre-compiled databases, and thus throughout the eukaryotic tree of life.

## Supplementary Material

gkad467_Supplemental_Files

## Data Availability

All data and software used in this study is open source. The ncOrtho algorithm is available on GitHub (https://github.com/BIONF/ncortho) or FigShare (https://doi.org/10.6084/m9.figshare.21679568.v1). The human miRNA orthologs are available in the Supplementary Data where we also supply a list of all genome assemblies which were used for the ortholog search.
