# Supplementary material for: ncOrtho: efficient and reliable identification of miRNA orthologs
Source: Nucleic Acids Res. 2023 Jun 1;51(13):e71. doi: 10.1093/nar/gkad467 (PMC10359484; doi:10.1093/nar/gkad467)
Supplement: gkad467_Supplemental_Files [file gkad467_supplemental_files.zip › ncOrtho_Supplementary_Figures_AR2.pdf]

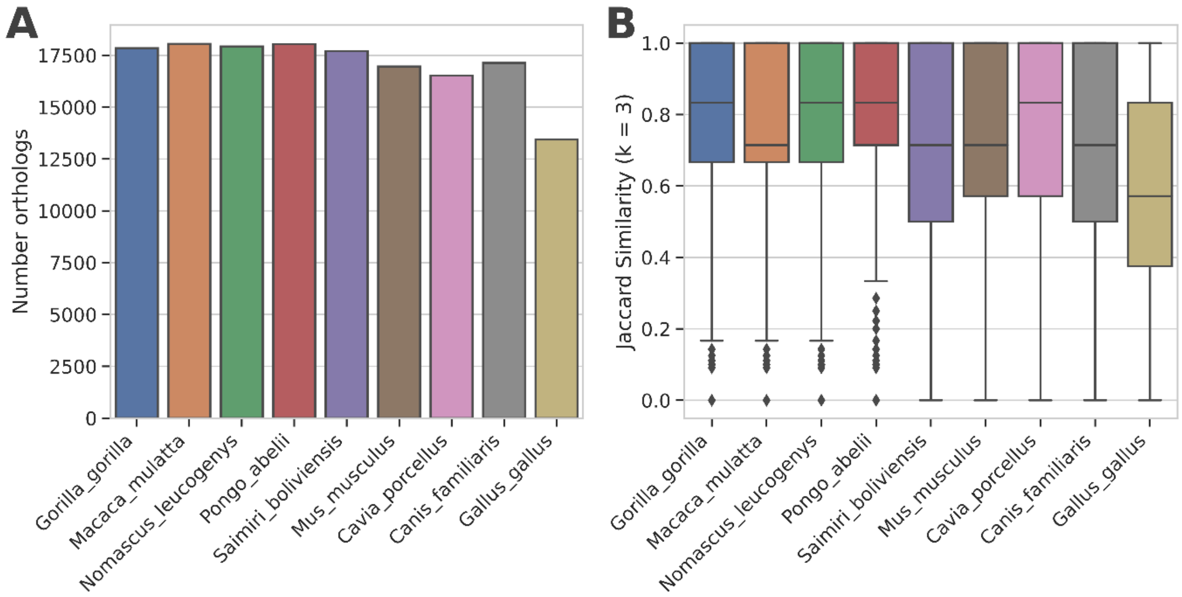

Fig. S1 Estimate of conserved microsynteny as calculated by ncCheck. Number of ortholog pairs between the reference (*Homo sapiens)* and the indicated species. B) Assessment of microsynteny conservation between the species from (A). In brief, for each ortholog pair of protein-coding genes in the reference and core species, we identify the k flanking protein-coding genes in each genome. We then calculate the the Jaccard similarity index between the two gene sets treating a gene in the reference set and in the core species set as shared if they have been identified as orthologs. The Jaccard similarity index is then used as a proxy for the extent of shared synteny.

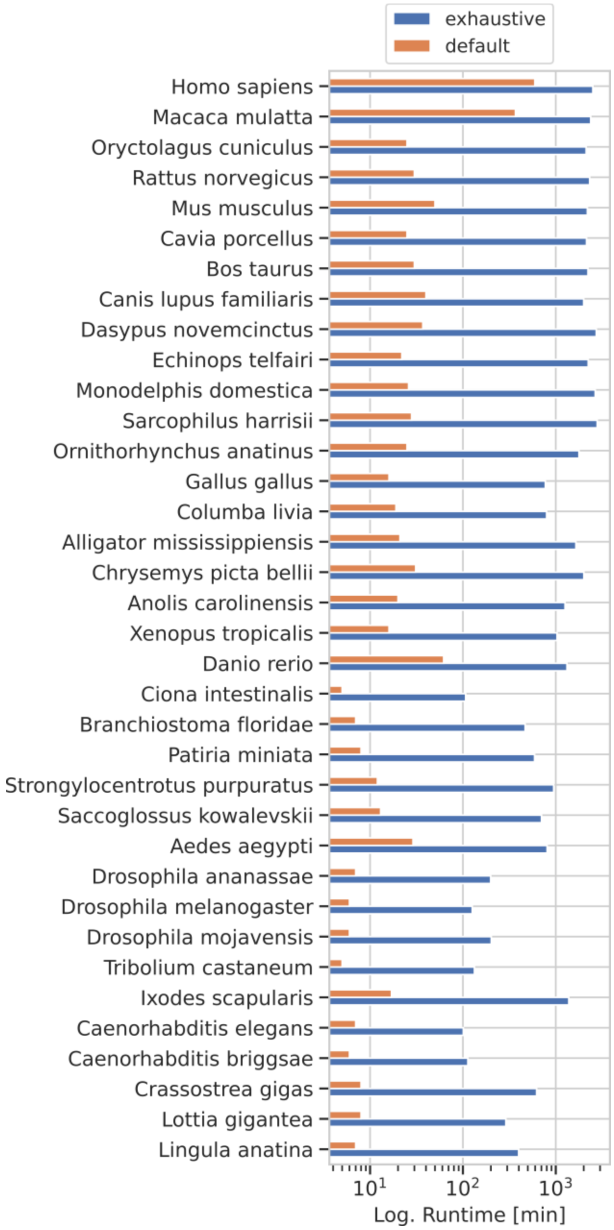

Figure S2: ncOrtho run time differences between exhaustive- and quick search. Bars represent, for each species, the mean run time of an ncOrtho ortholog search to 556 human miRNAs using the quick mode (orange) and the exhaustive mode (blue). Runtime (log scale) is given as wall clock time using 4 CPUs.

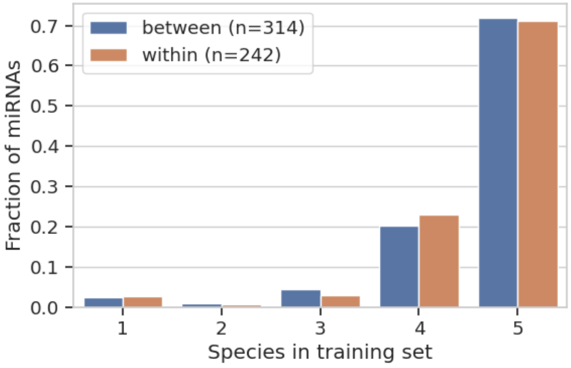

Fig. S3: Core ortholog group size distribution for miRNA genes located between and within protein genes. The histograms provide the fraction of miRNA genes within (orange; n=242) and between (blue; n=314) protein-coding genes with the indicated numbers of core species represented in the core ortholog group.

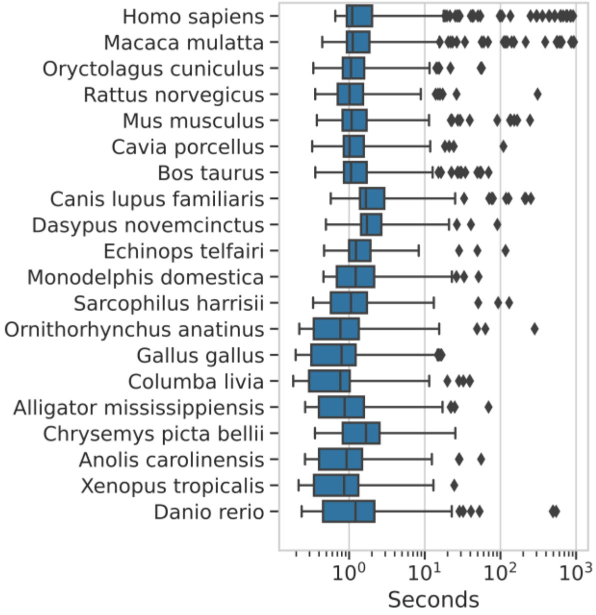

Fig. S4 Runtime of ncOrtho per miRNA. Ortholog search was performed for 556 human miRNAs in 20 vertebrate species. miRNA genes with longer runtimes are from families with a history of frequent duplications (e.g. Mir-430 or Mir-17)

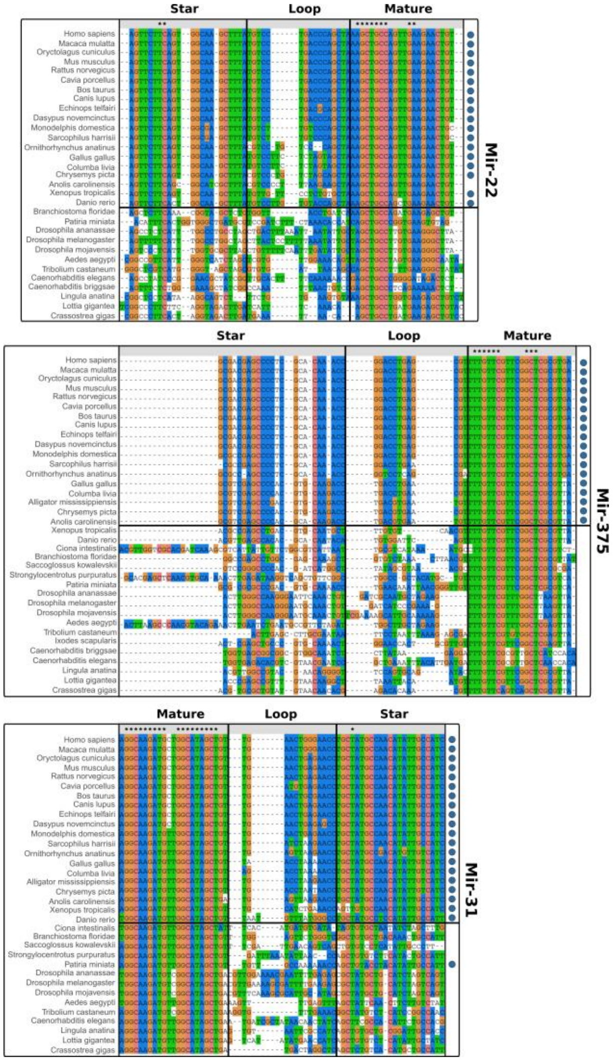

Figure S5: Sequence conservation patterns of miRNAs within vertebrates and across animals. The three multiple sequence alignments represent miRNAs with representatives both in vertebrates and invertebrates, according to MirGeneDB. The horizontal black ruler separates vertebrate species from invertebrates. The position of the mature, loop, and star regions of the miRNA are indicated above the alignment. Sequences identified as orthologs to the human miRNA by ncOrtho are indicated with a dot next to the sequence. The alignments reveal that the loop region, and in parts also the star region, are substantially more diverse in the invertebrates compared to the vertebrates. This explains why most of these orthologs are not identified as candidates during the CM-based search.

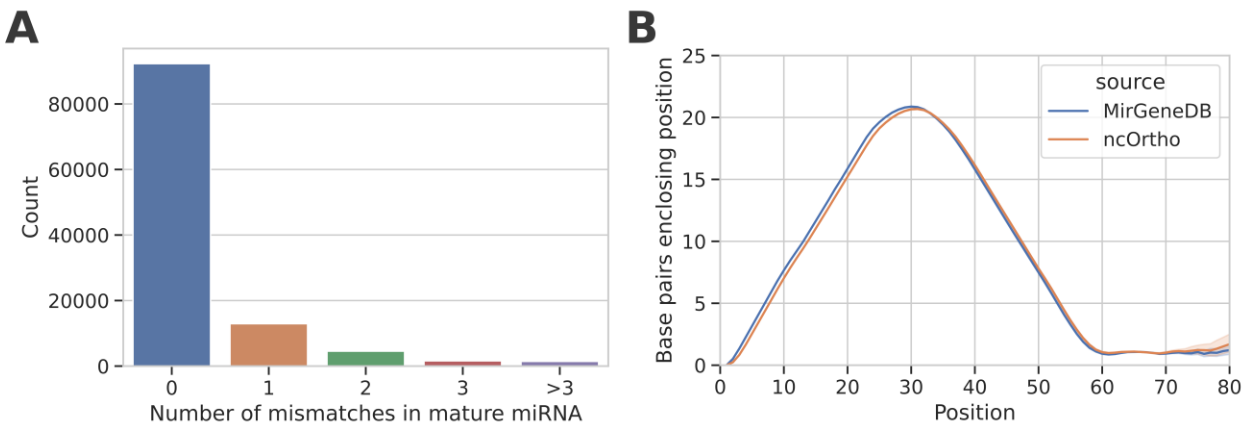

Fig. S6: Orthologs identified by ncOrtho in comparison to miRNA annotation guidelines defined by Ambros et al. (2003). A) Number of mismatches in alignments of the human mature miRNA and its ncOrtho orthologs. B) Mountainplot of miRNAs in MirGeneDB and miRNA orthologs predicted by ncOrtho. 95%-confidence interval is shown as shaded area. We limited the analysis to the first 80 nt of the predicted ortholog, which affects 198 out of 112,819 orthology assignments.

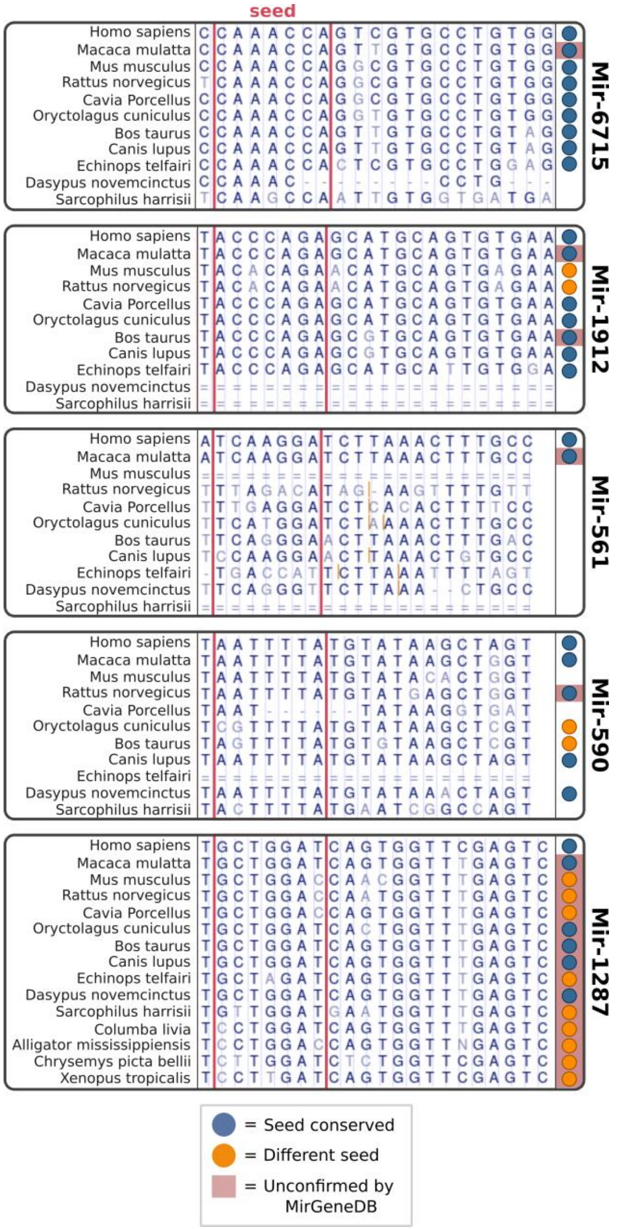

Figure S7: Multiple sequence alignment of a human miRNA locus across a collection of vertebrates. The human sequence in each alignment is the indicated mature human mirRNA. The aligned sequences represent the corresponding orthologous loci as extracted from the 100 vertebrates whole genome alignments provided via the UCSC Genome Browser. The red lines highlight the seed region. A dot identifies sequences that were identified as orthologs by ncOrtho and dot color indicates if the seed sequence is the same as in human (blue) or not (orange). A red background color indicates that the ortholog is not confirmed by MirGeneDB.

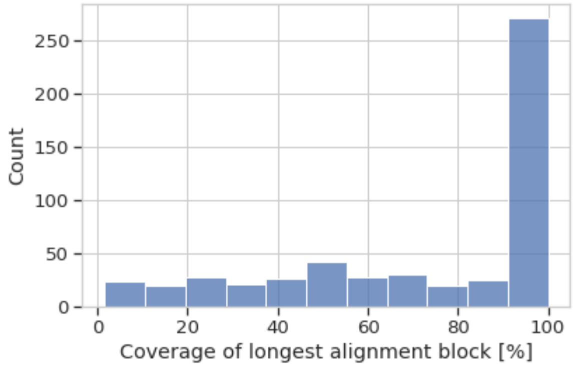

Fig. S8: Amount of human miRNA loci covered by continuous alignment blocks in the 100 Vertebrate track of the UCSC Genome Browser.

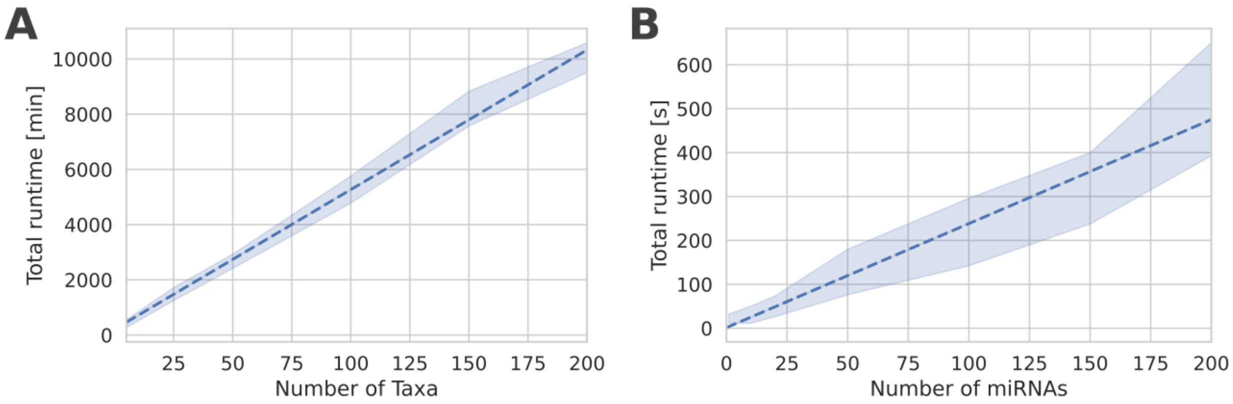

Figure S9: Run time of ncOrtho. (A) The CPU time increases linearly with the number of investigated species. We randomly compiled 10 taxon sets of the indicated sizes from the total of 402 vertebrate taxa. For each taxon set, we measured the run time (CPU time) for the ortholog search using 556 human miRNAs as input. The shaded area shows the 95% confidence interval of the resulting CPU times, and the dotted line a linear regression through the data. (B) The CPU time increases linearly with the number of reference miRNAs. We compiled 10 randomly selected miRNA subsets from a total of 556 human miRNAs. For each miRNA subset, we determined the runtime for the ortholog search in *M. musculus* 10 times.

See S10_Genelevel_PhyloProfile.pdf

Figure S10: Phylogenetic profile of 556 human miRNAs genes throughout the vertebrates. Green dots denote the number of co-orthologs detected. Dot color indicates whether the human seed sequence is conserved (blue) or not (orange).

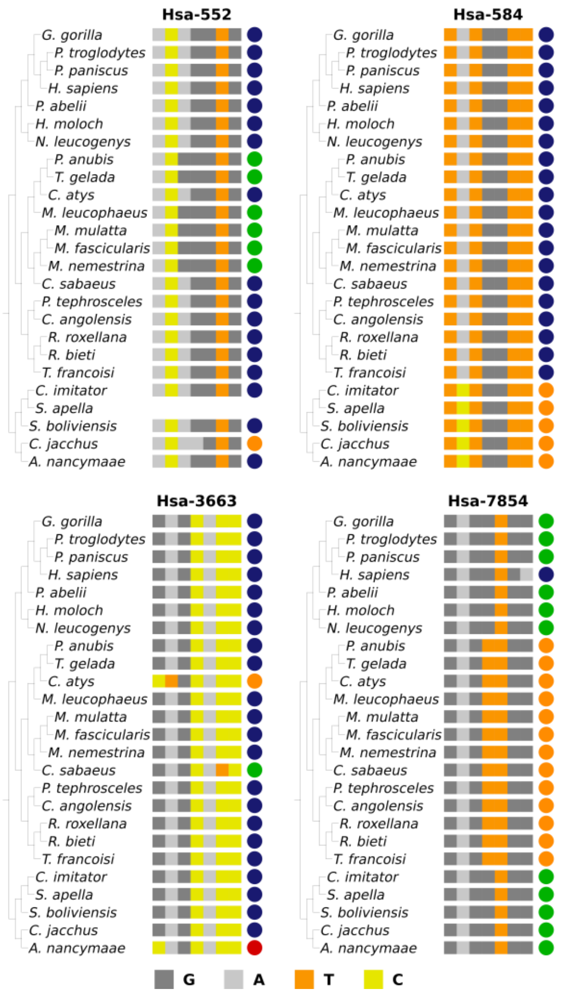

Figure S11: Selection of four seed sequence alignments for higher primate miRNA orthologs with a change in the seed sequence. The multiple sequence alignments are color coded to increase interpretability. A dot indicates that a sequence was identified as an ortholog to the human miRNA by ncOrtho. Orthologs sharing the same dot color have the same seed sequence. The data shows that seeds are highly conserved and individual to only few substitutions explain the seed sequence changes.

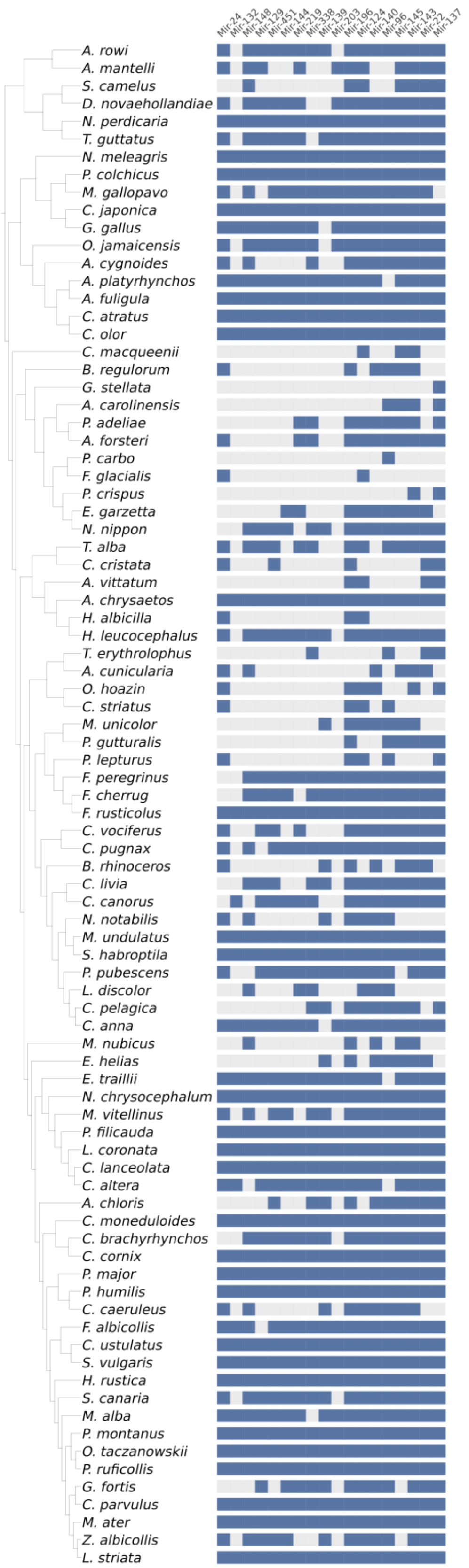

Mir-24
Mir-132
Mir-148
Mir-129
Mir-451
Mir-144
Mir-219
Mir-338
Mir-139
Mir-203
Mir-196
Mir-124
Mir-140
Mir-96
Mir-145
Mir-143
Mir-22
Mir-137
A. rowi
A. mantelli
S. camelus
D. novaehollandiae
N. perdicaria
T. guttatus
N. meleagris
P. colchicus
M. gallopavo
C. japonica
G. gallus
O. jamaicensis
A. cygnoides
A. platyrhynchos
A. fuligula
C. atratus
C. olor
C. macqueenii
B. regulorum
G. stellata
A. carolinensis
P. adeliae
A. forsteri
P. carbo
F. glacialis
P. crispus
E. garzetta
N. nippon
T. alba
C. cristata
A. vittatum
A. chrysaetos
H. albicilla
H. leucocephalus
T. erythrolophus
A. cunicularia
O. hoazin
C. striatus
M. unicolor
P. gutturalis
P. lepturus
F. peregrinus
F. cherrug
F. rusticolus
C. vociferus
C. pugnax
B. rhinoceros
C. livia
C. canorus
N. notabilis
M. undulatus
S. habroptila
P. pubescens
L. discolor
C. pelagica
C. anna
M. nubicus
E. helias
E. traillii
N. chrysocephalum
M. vitellinus
P. filicauda
L. coronata
C. lanceolata
C. altera
A. chloris
C. moneduloides
C. brachyrhynchos
C. cornix
P. major
P. humilis
C. caeruleus
F. albicollis
C. ustulatus
S. vulgaris
H. rustica
S. canaria
M. alba
P. montanus
O. taczanowskii
P. ruficollis
G. fortis
C. parvulus
M. ater
Z. albicollis
L. striata

Figure S12: Phylogenetic profiles of 18 miRNA families with sporadic absence in birds. The 18 miRNA families are represented in the columns, and each bird species is represented by a row in the matrix. Blue boxes indicate the representation of the mirRNA family by at least one ortholog in the corresponding species. The tree represents the phylogenetic relationships of the bird species according to the tree shown in Figure 6 of the main text. The data reveals that the observed presence / absence pattern of the 18 miRNA families in birds requires the assumption of multiple independent loss events.

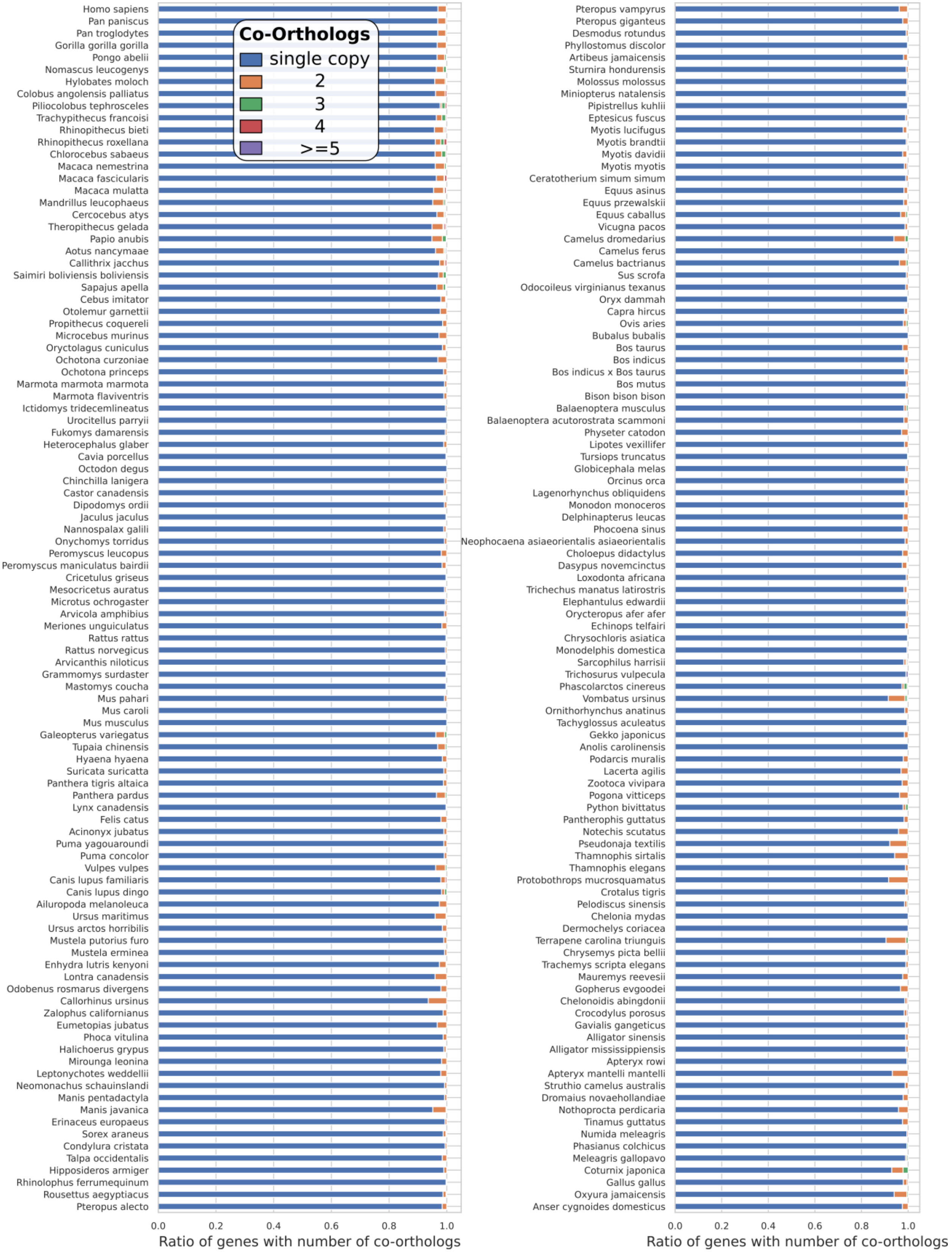
Co-Orthologs
single copy
2
3
4
>=5
Homo sapiens
Pan paniscus
Pan troglodytes
Gorilla gorilla gorilla
Pongo abelii
Nomascus leucogenys
Hylobates moloch
Colobus angolensis palliatus
Piliocolobus tephrosceles
Trachypithecus francoisi
Rhinopithecus bieti
Rhinopithecus roxellana
Chlorocebus sabaeus
Macaca nemestrina
Macaca fascicularis
Macaca mulatta
Mandrillus leucophaeus
Cercocebus atys
Theropithecus gelada
Papio anubis
Aotus nancymaae
Callithrix jacchus
Saimiri boliviensis boliviensis
Sapajus apella
Cebus imitator
Otolemur garnettii
Propithecus coquereli
Microcebus murinus
Oryctolagus cuniculus
Ochotona curzoniae
Ochotona princeps
Marmota marmota marmota
Marmota flaviventris
Ictidomys tridecemlineatus
Urocitellus parryii
Fukomys damarensis
Heterocephalus glaber
Cavia porcellus
Octodon degus
Chinchilla lanigera
Castor canadensis
Dipodomys ordii
Jaculus jaculus
Nannospalax galili
Onychomys torridus
Peromyscus leucopus
Peromyscus maniculatus bairdii
Cricetulus griseus
Mesocricetus auratus
Microtus ochrogaster
Arvicola amphibius
Meriones unguiculatus
Rattus rattus
Rattus norvegicus
Arvicanthis niloticus
Grammomys surdaster
Mastomys coucha
Mus pahari
Mus caroli
Mus musculus
Galeopterus variegatus
Tupaia chinensis
Hyaena hyaena
Suricata suricatta
Panthera tigris altaica
Panthera pardus
Lynx canadensis
Felis catus
Acinonyx jubatus
Puma yagouaroundi
Puma concolor
Vulpes vulpes
Canis lupus familiaris
Canis lupus dingo
Ailuropoda melanoleuca
Ursus maritimus
Ursus arctos horribilis
Mustela putorius furo
Mustela erminea
Enhydra lutris kenyoni
Lontra canadensis
Odobenus rosmarus divergens
Callorhinus ursinus
Zalophus californianus
Eumetopias jubatus
Phoca vitulina
Halichoerus grypus
Mirounga leonina
Leptonychotes weddellii
Neomonachus schauinslandi
Manis pentadactyla
Manis javanica
Erinaceus europaeus
Sorex araneus
Condylura cristata
Talpa occidentalis
Hipposideros armiger
Rhinolophus ferrumequinum
Rousettus aegyptiacus
Pteropus alecto
0.0
0.2
0.4
0.6
0.8
1.0
Ratio of genes with number of co-orthologs
Pteropus vampyrus
Pteropus giganteus
Desmodus rotundus
Phyllostomus discolor
Artibeus jamaicensis
Sturnira hondurensis
Molossus molossus
Miniopterus natalensis
Pipistrellus kuhlii
Eptesicus fuscus
Myotis lucifugus
Myotis brandtii
Myotis davidii
Myotis myotis
Ceratotherium simum simum
Equus asinus
Equus przewalskii
Equus caballus
Vicugna pacos
Camelus dromedarius
Camelus ferus
Camelus bactrianus
Sus scrofa
Odocoileus virginianus texanus
Oryx dammah
Capra hircus
Ovis aries
Bubalus bubalis
Bos taurus
Bos indicus
Bos indicus x Bos taurus
Bos mutus
Bison bison bison
Balaenoptera musculus
Balaenoptera acutorostrata scammoni
Physeter catodon
Lipotes vexillifer
Tursiops truncatus
Globicephala melas
Orcinus orca
Lagenorhynchus obliquidens
Monodon monoceros
Delphinapterus leucas
Phocoena sinus
Neophocaena asiaeorientalis asiaeorientalis
Choloepus didactylus
Dasypus novemcinctus
Loxodonta africana
Trichechus manatus latirostris
Elephantulus edwardii
Orycteropus afer afer
Echinops telfairi
Chrysochloris asiatica
Monodelphis domestica
Sarcophilus harrisii
Trichosurus vulpecula
Phascolarctos cinereus
Vombatus ursinus
Ornithorhynchus anatinus
Tachyglossus aculeatus
Gekko japonicus
Anolis carolinensis
Podarcis muralis
Lacerta agilis
Zootoca vivipara
Pogona vitticeps
Python bivittatus
Pantherophis guttatus
Notechis scutatus
Pseudonaja textilis
Thamnophis sirtalis
Thamnophis elegans
Protobothrops mucrosquamatus
Crotalus tigris
Pelodiscus sinensis
Chelonia mydas
Dermochelys coriacea
Terrapene carolina triunguis
Chrysemys picta bellii
Trachemys scripta elegans
Mauremys reevesii
Gopherus evgoodei
Chelonoidis abingdonii
Crocodylus porosus
Gavialis gangeticus
Alligator sinensis
Alligator mississippiensis
Apteryx rowi
Apteryx mantelli mantelli
Struthio camelus australis
Dromaius novaehollandiae
Nothoprocta perdicaria
Tinamus guttatus
Numida meleagris
Phasianus colchicus
Meleagris gallopavo
Coturnix japonica
Gallus gallus
Oxyura jamaicensis
Anser cygnoides domesticus
0.0
0.2
0.4
0.6
0.8
1.0
Ratio of genes with number of co-orthologs

Figure S13: Fraction of human miRNAs represented by 2 or more co-orthologs in 402 vertebrate species. Species are ordered from top to bottom according to increasing phylogenetic distance to humans

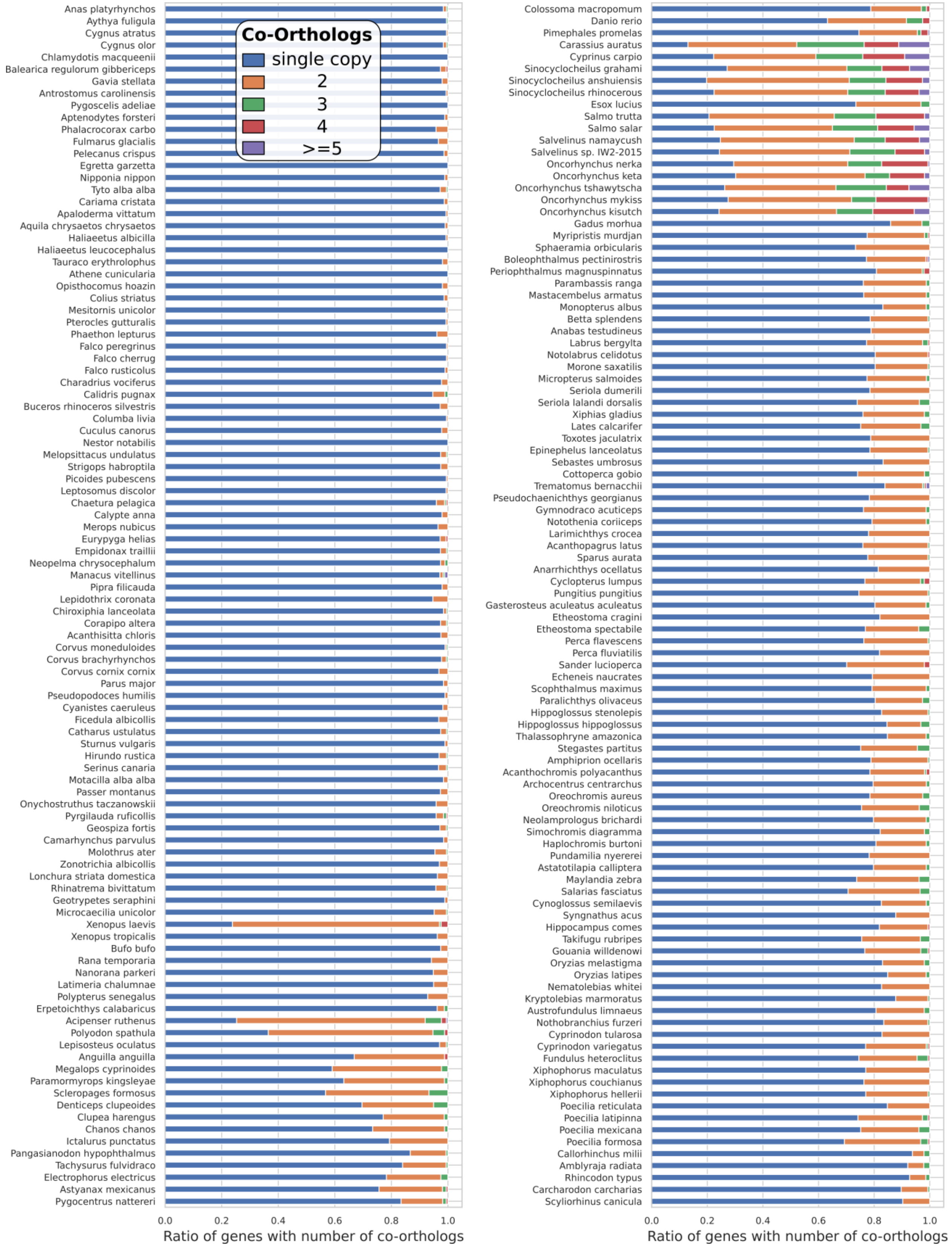
Co-Orthologs
single copy
2
3
4
>=5
Anas platyrhynchos
Aythya fuligula
Cygnus atratus
Cygnus olor
Chlamydotis macqueenii
Balearica regulorum gibbericeps
Gavia stellata
Antrostomus carolinensis
Pygoscelis adeliae
Aptenodytes forsteri
Phalacrocorax carbo
Fulmarus glacialis
Pelecanus crispus
Egretta garzetta
Nipponia nippon
Tyto alba alba
Cariama cristata
Apaloderma vittatum
Aquila chrysaetos chrysaetos
Haliaeetus albicilla
Haliaeetus leucocephalus
Tauraco erythrolophus
Athene cunicularia
Opisthocomus hoazin
Colius striatus
Mesitornis unicolor
Pterocles gutturalis
Phaethon lepturus
Falco peregrinus
Falco cherrug
Falco rusticolus
Charadrius vociferus
Calidris pugnax
Buceros rhinoceros silvestris
Columba livia
Cuculus canorus
Nestor notabilis
Melopsittacus undulatus
Strigops habroptila
Picoides pubescens
Leptosomus discolor
Chaetura pelagica
Calypte anna
Merops nubicus
Eurypyga helias
Empidonax traillii
Neopelma chrysocephalum
Manacus vitellinus
Pipra filicauda
Lepidothrix coronata
Chiroxiphia lanceolata
Corapipo altera
Acanthisitta chloris
Corvus moneduloides
Corvus brachyrhynchos
Corvus cornix cornix
Parus major
Pseudopodoces humilis
Cyanistes caeruleus
Ficedula albicollis
Catharus ustulatus
Sturnus vulgaris
Hirundo rustica
Serinus canaria
Motacilla alba alba
Passer montanus
Onychostruthus taczanowskii
Pyrgilauda ruficollis
Geospiza fortis
Camarhynchus parvulus
Molothrus ater
Zonotrichia albicollis
Lonchura striata domestica
Rhinatrema bivittatum
Geotrypetes seraphini
Microcaecilia unicolor
Xenopus laevis
Xenopus tropicalis
Bufo bufo
Rana temporaria
Nanorana parkeri
Latimeria chalumnae
Polypterus senegalus
Erpetoichthys calabaricus
Acipenser ruthenus
Polyodon spathula
Lepisosteus oculatus
Anguilla anguilla
Megalops cyprinoides
Paramormyrops kingsleyae
Scleropages formosus
Denticeps clupeoides
Clupea harengus
Chanos chanos
Ictalurus punctatus
Pangasianodon hypophthalmus
Tachysurus fulvidraco
Electrophorus electricus
Astyanax mexicanus
Pygocentrus nattereri
0.0
0.2
0.4
0.6
0.8
1.0
Ratio of genes with number of co-orthologs
Colossoma macropomum
Danio rerio
Pimephales promelas
Carassius auratus
Cyprinus carpio
Sinocyclocheilus grahami
Sinocyclocheilus anshuiensis
Sinocyclocheilus rhinocerous
Esox lucius
Salmo trutta
Salmo salar
Salvelinus namaycush
Salvelinus sp. IW2-2015
Oncorhynchus nerka
Oncorhynchus keta
Oncorhynchus tshawytscha
Oncorhynchus mykiss
Oncorhynchus kisutch
Gadus morhua
Myripristis murdjan
Sphaeramia orbicularis
Boleophthalmus pectinirostris
Periophthalmus magnuspinnatus
Parambassis ranga
Mastacembelus armatus
Monopterus albus
Betta splendens
Anabas testudineus
Labrus bergylta
Notolabrus celidotus
Morone saxatilis
Micropterus salmoides
Seriola dumerili
Seriola lalandi dorsalis
Xiphias gladius
Lates calcarifer
Toxotes jaculatrix
Epinephelus lanceolatus
Sebastes umbrosus
Cottoperca gobio
Trematomus bernacchii
Pseudochaenichthys georgianus
Gymnodraco acuticeps
Notothenia coriiceps
Larimichthys crocea
Acanthopagrus latus
Sparus aurata
Anarrhichthys ocellatus
Cyclopterus lumpus
Pungitius pungitius
Gasterosteus aculeatus aculeatus
Etheostoma cragini
Etheostoma spectabile
Perca flavescens
Perca fluviatilis
Sander lucioperca
Echeneis naucrates
Scophthalmus maximus
Paralichthys olivaceus
Hippoglossus stenolepis
Hippoglossus hippoglossus
Thalassophryne amazonica
Stegastes partitus
Amphiprion ocellaris
Acanthochromis polyacanthus
Archocentrus centrarchus
Oreochromis aureus
Oreochromis niloticus
Neolamprologus brichardi
Simochromis diagramma
Haplochromis burtoni
Pundamilia nyererei
Astatotilapia calliptera
Maylandia zebra
Salarias fasciatus
Cynoglossus semilaevis
Syngnathus acus
Hippocampus comes
Takifugu rubripes
Gouania willdenowi
Oryzias melastigma
Oryzias latipes
Nematolebias whitei
Kryptolebias marmoratus
Austrofundulus limnaeus
Nothobranchius furzeri
Cyprinodon tularosa
Cyprinodon variegatus
Fundulus heteroclitus
Xiphophorus maculatus
Xiphophorus couchianus
Xiphophorus hellerii
Poecilia reticulata
Poecilia latipinna
Poecilia mexicana
Poecilia formosa
Callorhinchus milii
Amblyraja radiata
Rhincodon typus
Carcharodon carcharias
Scyliorhinus canicula
0.0
0.2
0.4
0.6
0.8
1.0
Ratio of genes with number of co-orthologs

Figure S13 (continued): Fraction of human miRNAs represented by 2 or more co-orthologs in 402 vertebrate species. Species are ordered from top to bottom according to increasing phylogenetic distance to humans

See S14_Phylogenetictree_with_CarlitoSyrichta.pdf

Figure S14: Vertebrate species tree reconstructed from 556 pre-miRNA alignments including *Carlito syrichta*. Branch labels denote percent bootstrap support.

See S15_Vertebrate_MLtree.pdf

Figure S15: Vertebrate species tree reconstructed from 556 pre-miRNA alignments excluding *Carlito syrichta*.

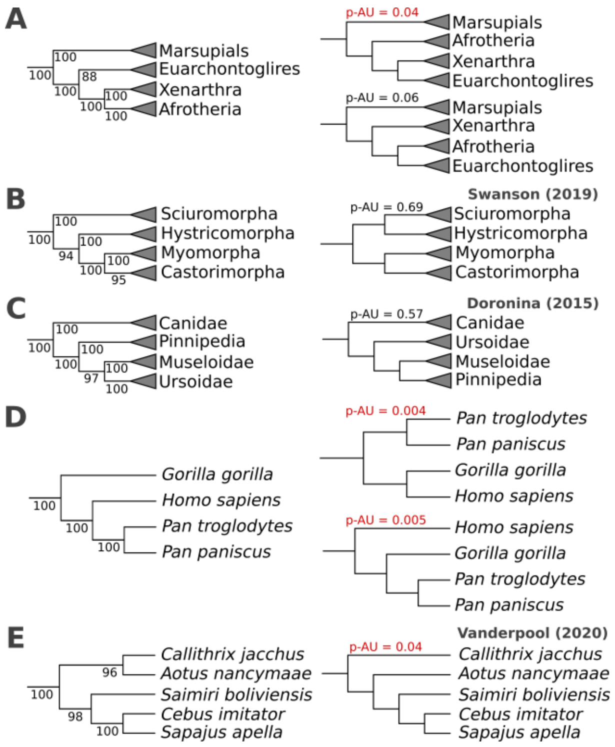

Figure S16: Alternative hypotheses for clades where ILS was claimed to interfere with the species tree reconstruction. The topology on the left represents the corresponding subtree of Figure 6 in the main text. Topologies on the right are alternative hypotheses that have been discussed in the literature. See main text for further details. Results of the approximately unbiased (AU) test (Shimodaira, 2002) are given for alternative tree topologies and are marked in red if significant on the 5% level. Branch lengths are not drawn to scale. Branch labels denote percent bootstrap support.

Table S1: NCBI Accession Number of Assemblies used in the study.

Table S2: Orthologs to 556 human miRNA genes across 402 vertebrate species detected by ncOrtho.

Table S3: Point of origin and number of losses of human miRNA families.
