# Supplementary figures and images for: ncOrtho: efficient and reliable identification of miRNA orthologs
Source: Nucleic Acids Res. 2023 Jun 1;51(13):e71. doi: 10.1093/nar/gkad467 (PMC10359484; doi:10.1093/nar/gkad467)

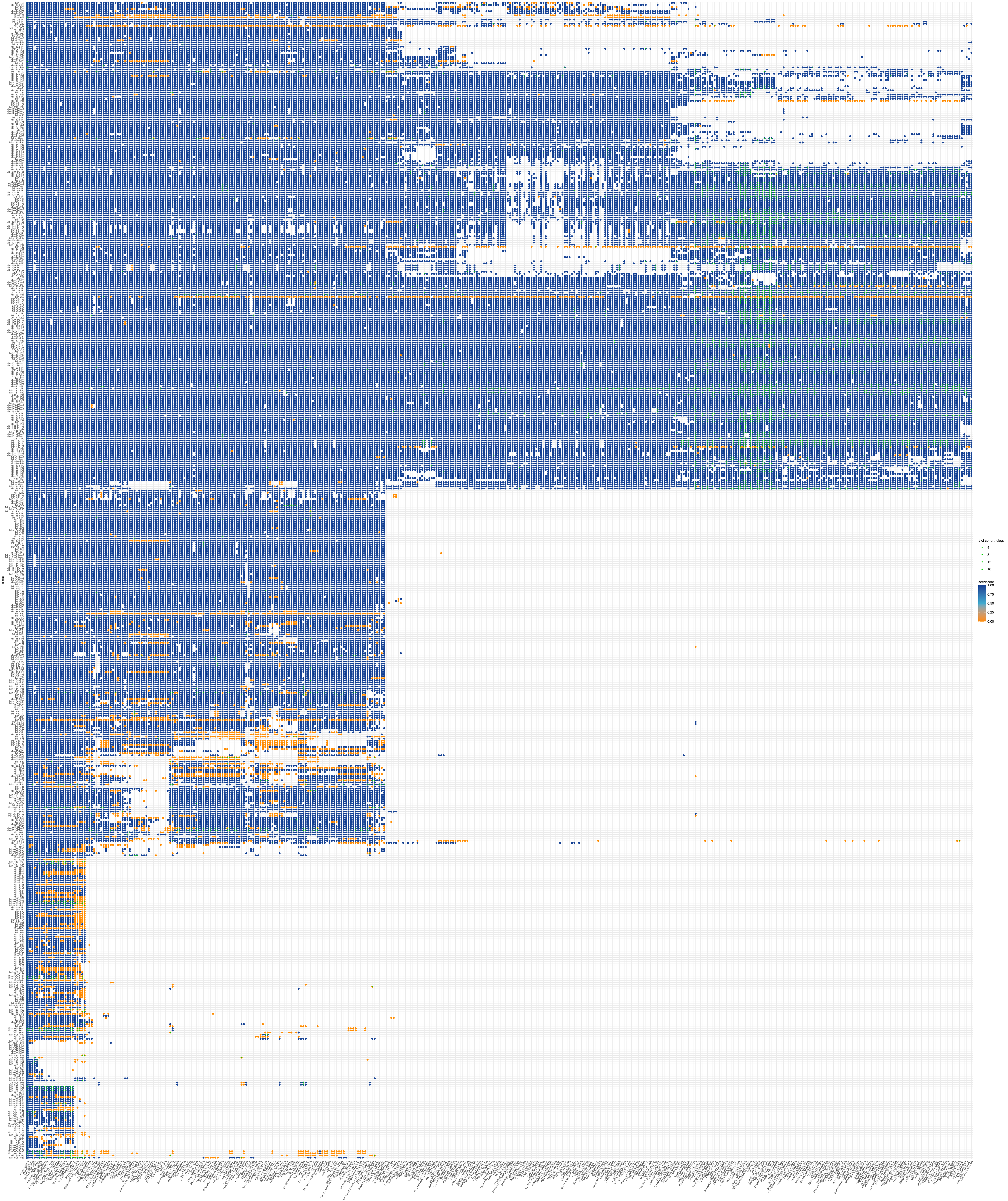

Supplement: gkad467_Supplemental_Files [file gkad467_supplemental_files.zip › S10_Genelevel_PhyloProfile.pdf]

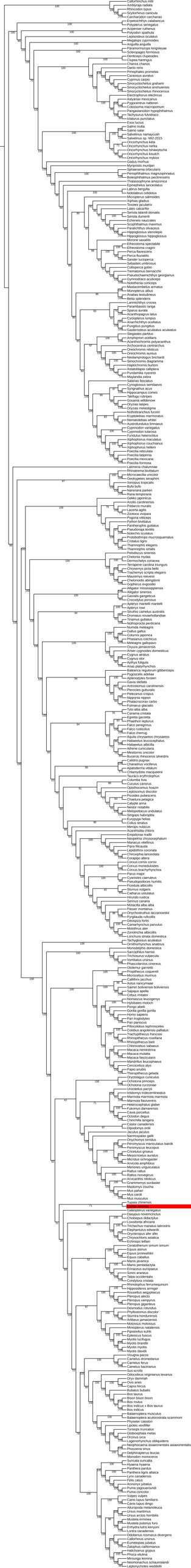

Supplement: gkad467_Supplemental_Files [file gkad467_supplemental_files.zip › S14_Phylogenetictree_with_CarlitoSyrichta.pdf]

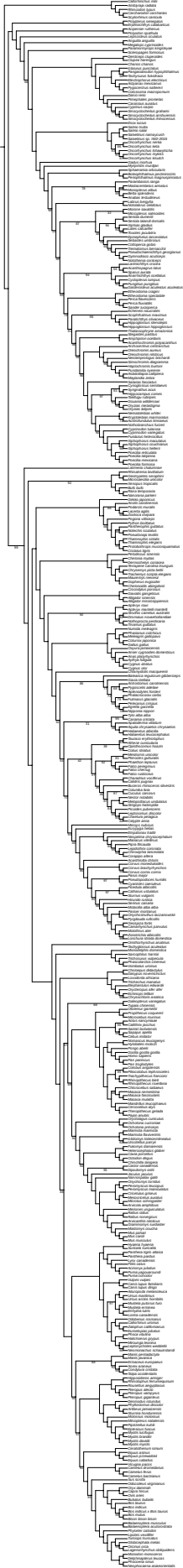

Supplement: gkad467_Supplemental_Files [file gkad467_supplemental_files.zip › S15_Vertebrate_MLtree.pdf]
